# Coupling Molecular Spin Qubits with 2D Magnets for Coherent Magnon Manipulation

**DOI:** 10.1021/acs.nanolett.5c01937

**Published:** 2025-06-17

**Authors:** Sourav Dey, Gonzalo Rivero-Carracedo, Andrei Shumilin, Carlos Gonzalez-Ballestero, José J. Baldoví

**Affiliations:** † Instituto de Ciencia Molecular (ICMol), 201469 Universitat de Valencia, c/Catedrático José Beltrán, 2, Paterna 46980, Spain; ‡ Washington State University, Pullman, Washington 99164, United States; § Institute for Theoretical Physics and Vienna Center for Quantum Science and Technology, TU Wien, 1040 Vienna, Austria

**Keywords:** spin qubit, magnonics, coherence, 2D materials, first-principles

## Abstract

Magnonics is an emerging field widely considered as a paradigm shift in information technology that uses spin waves for data storage, processing, and transmission. However, the coherent control of spin waves in 2D magnets still remains a challenge. Herein, we investigate the interplay between molecular spins and magnons in hybrid heterostructures formed by titanocene bis­(cyclooctatetraenyl) [CpTi­(cot)] and vanadyl phthalocyanine (VOPc) spin qubits deposited on the surface of the air-stable 2D van der Waals ferromagnet CrSBr using first principles. Our results show that different molecular rotation configurations significantly impact on qubit relaxation time and alter the magnon spectra of the underlying 2D magnet, allowing the chemical coherent control of spin waves in this material. We predict the feasibility of an ultrafast magnon-qubit interface with minimized decoherence, where exchange coupling plays a crucial role. This work opens new avenues for hybrid quantum magnonics, enabling selective tailoring through a versatile chemical approach.

## Graphical abstract

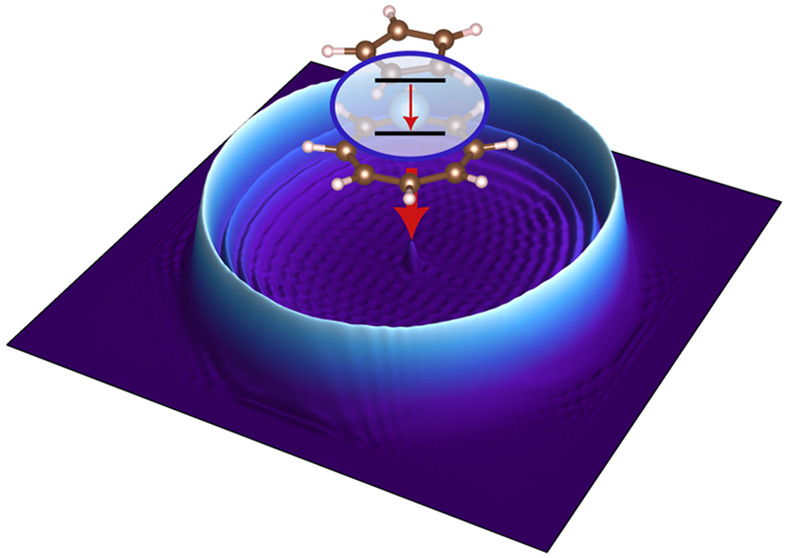

Magnonics is a rapidly growing field of research that deals with the storage, processing, and transmission of information based on the use of spin waves (SWs), i.e., collective magnetic excitations in magnetic materials, instead of electric charges. This novel technology provides an alternative to electronics, enabling the development of nanoscale magnonic devices with lower power consumption and higher frequencies.^1,2^ The recently born field of quantum magnonics^3^ aims at exploring the potential of SWs—and their quanta, magnons—as components in hybrid quantum technologies. This is due to SWs’ unconventional properties such as strong nonlinearity, tunability, or the ability to couple to almost every other degree of freedom,^4–6^ which can be easily tailored when compared to other quantum particles such as phonons or photons. Similar to the latter, the interest of magnons for quantum platforms relies on the ability to manipulate and control their coupling to local quantum nodes, that is, qubits.^7^ Several works have proposed methods to achieve this with superconducting qubits or solid-state spins, but they all focus on bulky ferrimagnetic samples.^8–13^ The high damping and low scalability of these systems has sparked an active search for new platforms to implement the basic unit of hybrid quantum magnonics: a qubit-magnon interface.^14^

The discovery of long-range magnetic ordering in two-dimensional (2D) materials provides an unprecedented opportunity to study SWs on this class of systems. Among the family of layered van der Waals (vdW) magnetic materials, those that retain ferromagnetic order down to the monolayer limit such as CrI_3_,^15^ Cr_2_Ge_2_Te_6_,^16^ CrSBr,^17^ Fe_3_GeTe_2_,^18^ or Fe_3_GaTe_2_,^19^ are at the forefront in research. These 2D magnets exhibit numerous advantages given that they represent the limit of miniaturization, have high flexibility, can be tuned through vdW stacking, and are compatible with silicon technology.^20,21^ In this regard, a promising and unexplored route is the creation of hybrid molecular/2D heterostructures formed by spin qubits and 2D magnetic materials for coherent magnon control. Recently, some works have focused on the manipulation of SWs in bulk counterparts, mostly in Yttrium Iron Garnet (YIG), especially using nitrogen-vacancy (NV) centers, as they can be interfaced with other widely used excitations (microwave photons or phonons) with high quantum cooperativities and – crucially– they can be optically initialized at room temperature.^12,22,23^ However, the manipulation of SWs by coupling them to a spin qubit on 2D magnetic materials has not been reported to the best of our knowledge.

Among qubits, molecular spin qubits are particularly interesting. These are two-level systems based on coordination complexes of paramagnetic metal ions, which have been broadly investigated, and deposited on different substrates, showing strong hybridization with them.^24–26^ In contrary to electron-spin based qubits, such as impurities in silicon^27^ or nitrogen vacancies in diamond,^28,29^ molecular spin qubits can couple with the substrate via magnetic exchange, which is much stronger than dipole–dipole interactions, allowing a more pronounced coupling to magnons. In addition, they exhibit higher tunability, i.e. different coordination geometries and ligands, thus opening new possibilities for the versatile coherent control of SWs.

In this work, we investigate the magnon emission – induced by a molecular spin qubit relaxation– in two hybrid molecular/2D magnetic heterostructures, formed by (i) [CpTi­(cot)] (titanocene bis­(cyclooctatetraenyl)) and (ii) VOPc (vanadyl phthalocyanine), deposited on the surface of the 2D ferromagnet CrSBr. Both molecules act as S = 1/2 qubits and have already been tested on different surfaces, preserving their spin state.^30–33^ This has allowed them to efficiently tune superconductivity and give rise to Yu-Shiba-Rusinov states in superconducting lead.^34^ On the other hand, CrSBr can be exfoliated down to the monolayer limit, exhibiting air-stability, high Curie temperature (T_C_ ∼ 146 K) and high energy magnons,^35–42^ which can be selectively tuned by strain, deposition of sublimable organic molecules or gas species.^43–45^ Through first-principles calculations, we analyze the effects of these molecular spin qubits on the structural, electronic and magnetic properties of CrSBr monolayer, and particularly in terms of their coupling with magnons. This work fosters the exploration of novel frontiers at the spinterface regarding the coherent manipulation of SWs in 2D magnetic materials.

CrSBr is a layered material that crystallizes in an orthorhombic structure and *Pmmn* space group. The Cr atoms reside in a distorted octahedral coordination environment and they are linked via S and Br atoms along *a* axis and via S along the *b* and diagonal *ab* directions (see Figure 1a, b). According to our calculations, the lattice parameters of CrSBr monolayer are a = 3.545 Å and b = 4.735 Å, in close agreement with previous reports.^46,47^ As molecular counterparts, we use (i) [CpTi­(cot)], which is an organometallic complex in which the metal atom is sandwiched between an eight-membered ring, η^8^-cyclooctatetraene (cot^2–^), and a five-membered ring, η^5^-cyclopentadienyl (Cp^–^),^48^ and (ii) VOPc, which is a nonplanar metal-phthalocyanine complex where the vanadyl ion (VO^2+^) is coordinated to the four nitrogen atoms of the phthalocyanine (Pc^2–^) ring (see Figure 1c, d).^49^

**Figure 1:**
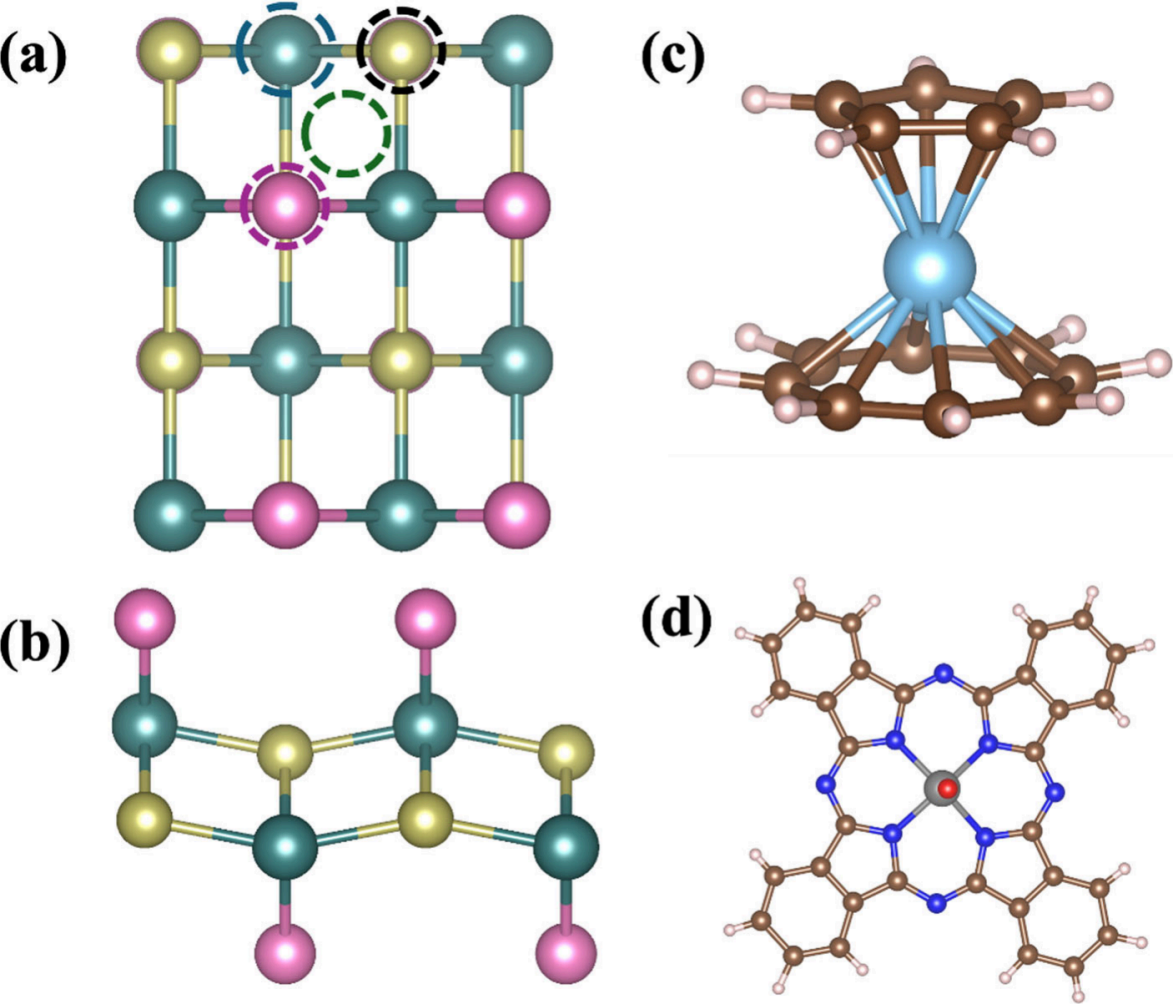
(a) Top view and (b) side view of CrSBr monolayer. The four adsorption sites such as top of Cr, Br, S, and hollow are indicated with blue, purple, orange, and green circles, respectively. Molecular structure of (c) [CpTi­(cot)] and (d) VOPc. Color code: Cr (green), Br (pink), S (yellow), Ti (cyan), V (silver gray), O (red), N (blue), C (brown), and H (white).

First, we study [CpTi­(cot)] and VOPc molecules in gas phase to determine their electronic structures. DFT calculations reveal the splitting of the 3d orbitals into nonbonding, bonding and antibonding sets, in both molecules. The nonbonding *d*_*z2*_ orbital in [CpTi­(cot)] and *d*_*xy*_ orbital in VOPc are the singly occupied molecular orbitals (SOMO), which are the magnetic orbitals in each molecule (see Figures S1 and S2 in the Supporting Information for details).

To investigate the effect of qubit adsorption on the magnetic properties of CrSBr, we design two heterostructures, namely [CpTi­(cot)]@CrSBr and VOPc@CrSBr, using 4 × 4 and 6 × 6 CrSBr supercells, respectively. In order to consider the molecular configuration on the substrate, we study three different orientations for [CpTi­(cot)]: *standing*_*cot*_, *standing*_*Cp*_, and *lying* (Figures 2 (top) and **S3** (top)). On the other hand, we study two possible VOPc orientations: *oxygen-up* and *oxygen-down* (Figures 2 (bottom) and **S3** (bottom)). Four different adsorption sites on CrSBr monolayer for both systems are considered, placing the metal atom of each qubit sitting on top of Br, Cr, S and hollow, as shown in Figure 1a.

**Figure 2:**
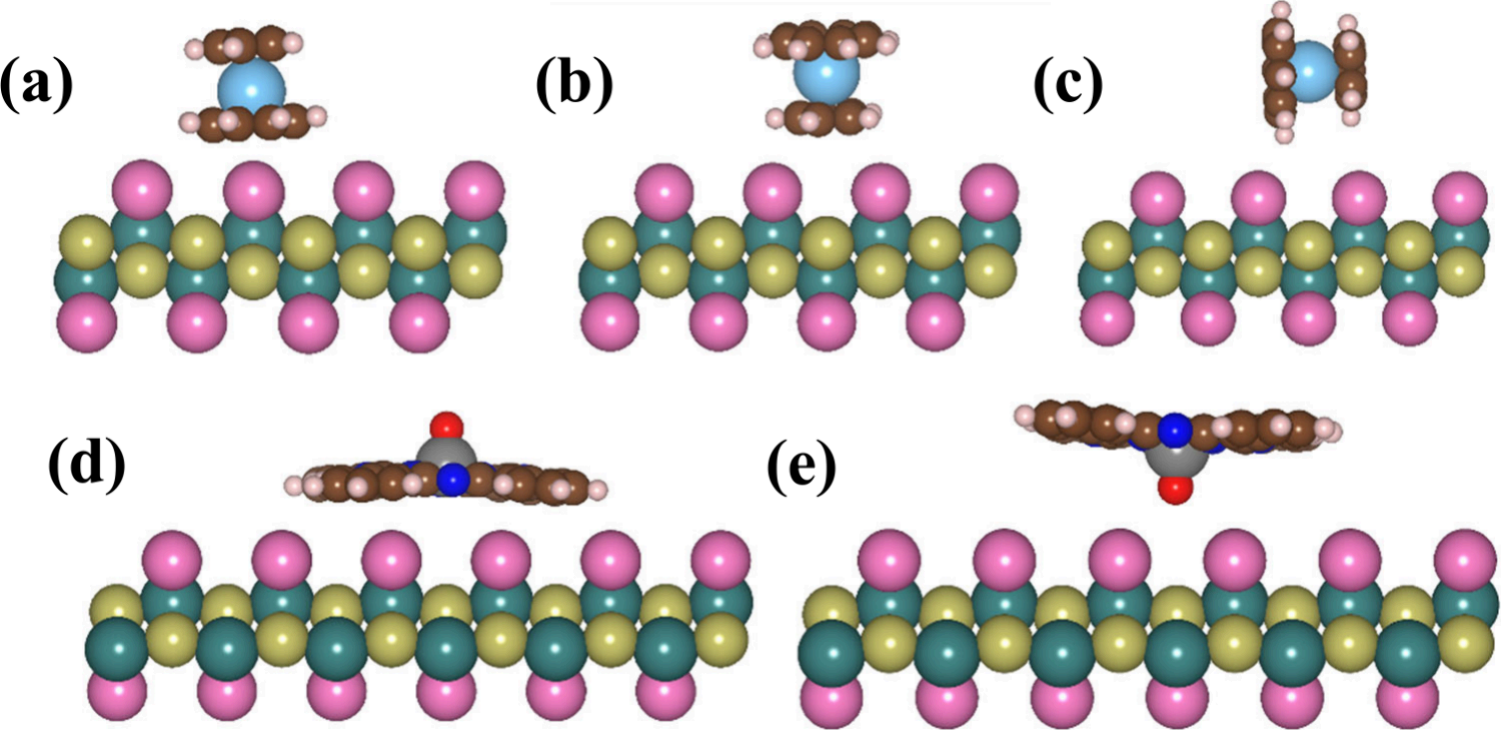
(Top) Side views of the optimized adsorption geometries of [CpTi­(cot)] on the most stable site of CrSBr in three orientations: (a) *standing*_*cot*_, (b) *standing*_*Cp*_, and (c) *lying*. (Bottom) Side views of the optimized adsorption geometries of VOPc on the most stable site of CrSBr, showing (d) *oxygen-up* and (e) *oxygen-down* configurations.

After a full optimization of the atomic coordinates, the calculated equilibrium distance between [CpTi­(cot)] and CrSBr are in the range 2.75–3.09 Å depending on the orientation (see Figure S4 and Table S1). Note that, among the three orientations of [CpTi­(cot)], *standing*_*cot*_ is the closest case (2.75 Å), exhibiting stronger interaction with the substrate compared to *standing*_*Cp*_ and *lying* orientations. In the case of VOPc, the largest interaction is observed for the *oxygen-up* orientation, given that the aromatic ring of the Pc is parallel to the surface. This allows a larger hybridization when compared to *oxygen-down* (Table S2). The molecule–substrate distance is around 3.0 Å, which also indicates a physisorption process. Subsequently, we calculate the adsorption energy (*E*_ads_) to compare the stability of the different heterostructures. Our calculations indicate that the *standing*_*cot*_ orientation (*E*_ads_ = −0.94 eV) is the most stable one for [CpTi­(cot)], followed by *standing*_*Cp*_ (*E*_ads_ = −0.67 eV) and *lying* (*E*_ads_ = −0.53 eV) orientations, respectively. All these magnitudes reveal a physisorption process governed by vdW interactions, mainly via dipole–dipole and π–surface interaction. In general, the difference in *E*_ads_ for both adsorption sites and orientations is small, which limits the prediction of a preferential configuration (Table S3). This is consistent with the observed behavior for [CpTi­(cot)] on Au(111) surface.^30^ For VOPc, the computed adsorption energy is 0.7 eV larger than that of [CpTi­(cot)] (Table S4). In particular, the *oxygen-up* orientation on top of the Cr site is found to be the most stable heterostructure (*E*_ads_ = −1.65 eV, Table S4). In contrast, for *oxygen-down* orientation, the adsorption energy was estimated to be 1.1 eV smaller compared to *oxygen-up* orientation (Table S4). Like [CpTi­(cot)], the adsorption energy differs by less than 0.1 eV for the four adsorption sites (Table S4).

Then, we perform a Bader charge transfer analysis^50^ to study the electron density flow at the interface for the most stable adsorption site (See section 3.2 of Supporting Information for details). Our calculations evidence the transfer of electrons from the molecule to the substrate for all molecular orientations and adsorption sites. For [CpTi­(cot)], the largest charge transfer (0.46e) is estimated for *standing*_*cot*_ orientation (which presents the largest adsorption energy), whereas for VOPc, a charge transfer of 0.24e is obtained for the *oxygen-up* orientation. The charge density differences (CDD) are reported in Figures S5 – S7.

Regarding the magnetic moments of the metal centers, in the case of [CpTi­(cot)]@CrSBr, we determine a 0.36 μ_B_ reduction in the magnetization of Ti, while the total magnetization of Cr in the substrate increases by 0.19 μ_B_ due to electron delocalization from the molecule to the substrate (Figures S8 - S10). To minimize spin delocalization, larger cyclic polyene ligands with greater surface area and weaker metal–ligand interactions should be used, as demonstrated by Sessoli and co-workers in [FluTi­(cot)], where the Ti atom retains its charge and spin on an Au surface.^51^ For VOPc, the magnetization of V remains almost unperturbed in both *oxygen-up* and *oxygen-down* orientations, and consequently, shows negligible spin delocalization with the substrate (Figures S5, S8, and S11). We also determined the effect of molecular adsorption on the electronic structure. A detailed explanation of the projected density of states is available in section 3.3 of Supporting Information.

Then, we investigate the effect of the adsorption of the qubit molecule on CrSBr magnetic exchange interactions. Magnetic couplings in pristine CrSBr are usually modeled by the three nearest-neighbor exchange interactions, namely *J*_1_, *J*_2_, and *J*_3_ (Figure 3a). *J*_1_ represents the magnetic exchange between Cr atoms along the *a* axis, *J*_2_ denotes the exchange interaction along the *ab* diagonal direction and *J*_3_ accounts for the exchange interaction along the *b* crystallographic direction (see characteristic angles in Table S5 and Figure S19 in Supporting Information). The adsorption of the molecular spin qubits on the 2D magnet leads to an additional exchange (*J*_4_), which takes place between the spin of the molecule and nearest-neighbors in the substrate (Figure 3b). These exchange interactions are determined by mapping the energies of different magnetic spin configurations (see Figures S20 and S21) into an isotropic Heisenberg Hamiltonian of the form:

1 $$Ĥ=-\underset{ij}{\sum }J_{ij}{ŝ}_{i}{ŝ}_{j}$$

where *J*_*ij*_ represents the exchange interaction between two different spins (*s02*_*i*_ and *s02*_*j*_). The spin configurations include different qubit spin orientations relative to CrSBr magnetization, whose stability indicates that partial charge transfer between qubit and the substrate does not preclude its pseudospin-1/2 description. Additionally, the qubit energy is calculated as *E*_*Q*_ = 2*S*_*Cr*_ *S*_*Q*_ *N*_*N*_ *J*_4_, where *S*_*Cr*_ represents the spin of Cr, which is 3/2; *S*_*Q*_ the spin of the qubit, which is 1/2 for both molecules; and *N*_*N*_ the number of nearest-neighbors Cr atoms to the spin qubit. The dipole–dipole interaction of the spin qubit with CrSBr (*E*_*dd*_) was calculated from optimized atomic coordinates (see section 5 of Supporting Information for details). The estimated magnetic exchange interactions for *J*_*1*_-*J*_*4*_, qubit energies and dipole–dipole interactions are reported in Table 1.

**Table 1:** Isotropic Magnetic Exchange Parameters (meV), Qubit Energies (*E*_*Q*_ (meV)), and Dipole–Dipole Energies (*E*_*dd*_ (meV)) for Different Molecular Orientations of [CpTi­(cot)] and VOPc on CrSBr^a^

		[CpTi(cot)]			VOPc	
	CrSBr	*standing_cot_*	*standing*_*Cp*_	*lying*	*oxygen-up*	*oxygen-down*
*J*_*1*_ *, meV*	2.93	2.82	2.77	2.77	2.66	2.65
*J*_*2*_ *, meV*	3.55	3.59	3.60	3.59	3.55	3.55
*J*_*3*_ *, meV*	2.33	2.78	2.68	2.72	2.05	2.00
*J*_*4*_ *, meV*		0.60	0.26	0.03	–0.02	0.01
*E*_*Q*_ *, meV*		1.80	0.78	0.08	0.14	0.02
*E*_*dd*_ *, meV*		0.02	0.01	0.01	0.02	0.01

a *J*_*1*_-*J*_*3*_ for pristine CrSBr are also reported for comparison.

**Figure 3:**
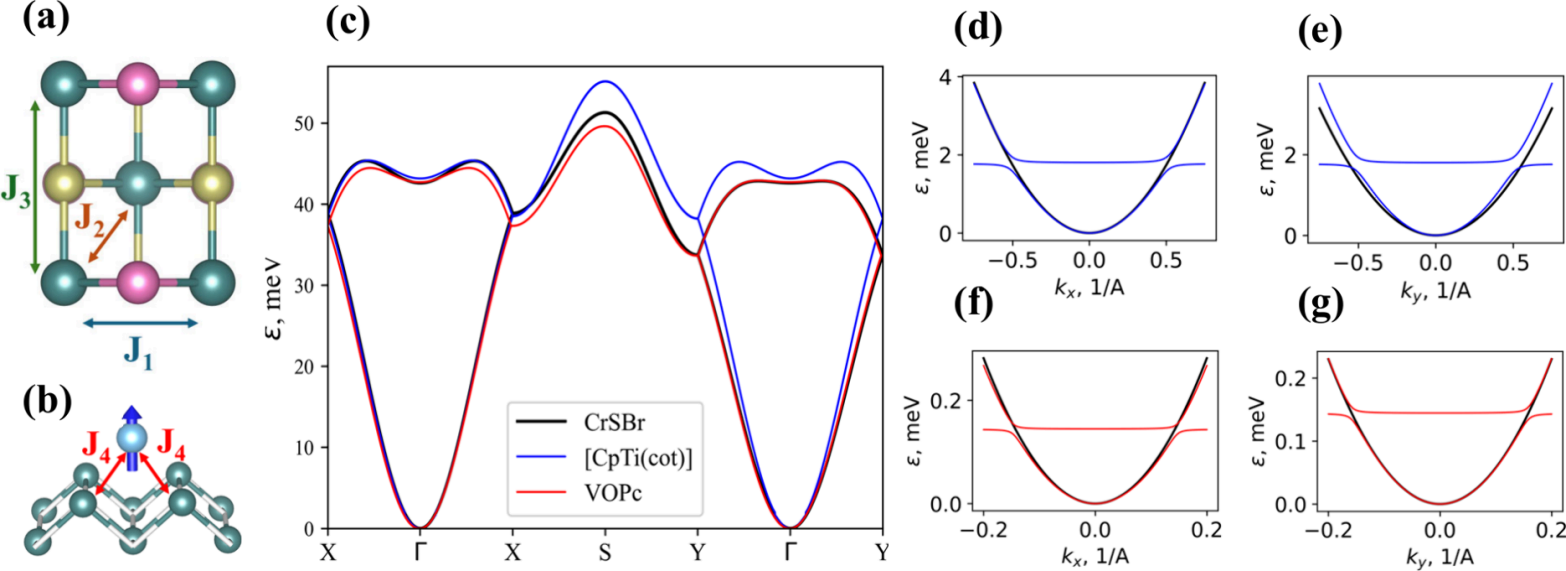
Schematic representation of the nearest-neighbor exchange interactions (a) *J*_*1*_, *J*_*2*_, and *J*_*3*_ for CrSBr and (b) *J*_*4*_ between molecular spin and the nearest Cr atoms from the substrate. (c) The spectrum of acoustical and optical magnons in pristine CrSBr and in the [CpTi­(cot)]@CrSBr and VOPc@CrSBr heterostructures with unfolded bands. (d-g) The spectrum of low-energy magnons near Γ-point for [CpTi­(cot)]@CrSBr (d,e) and VOPc@CrSBr (f,g) the blue/red lines represent the hybridized bands of acoustic magnons and qubits spins while black lines show acoustic magnons in pristine CrSBr.

The calculated magnetic exchanges for pristine CrSBr are 2.93 meV (*J*_1_), 3.55 meV (*J*_2_), and 2.33 (*J*_3_) meV and agree very well with experimental values.^52^ These exchange interactions are strongly modified in the [CpTi­(cot)]@CrSBr hybrid heterostructure compared to the VOPc one. The strongest effect is a 19% increase of *J*_3_ by the adsorption of [CpTi­(cot)] in the *standing_cot_* orientation. This change in magnetic exchange after adsorption stems from (i) substrate distortion, (ii) charge transfer from molecule to substrate, and (iii) molecule–substrate exchange interaction (see Table S7 and Supporting Information for details). On the other hand, *J*_4_ depends on both the orientation of the molecule and adsorption site on CrSBr, as we show in Table 1. According to our simulations, *J*_4_ is 0.60 meV for *standing*_*cot*_ orientation of [CpTi­(cot)] and −0.02 meV for *oxygen-up* orientation of VOPc, which corresponds to antiferromagnetic CrSBr-VOPc exchange interaction. The molecule–substrate exchange interaction can proceed via (i) direct exchange between the metal center and Cr 3d orbitals, leading to ferromagnetic coupling, or (ii) indirect exchange mediated through ligand orbitals, resulting in antiferromagnetic coupling.^53,54^ Our results show that the nature of this exchange interaction depends critically on both the position and orientation of the molecule on the surface. Notably, in all cases *E*_*Q*_ is much larger than *E*_*dd*_, showing a good interplay between the molecular spin qubits and the magnetic properties of CrSBr.

The magnon spectra for [CpTi­(cot)] (*standing*_*cot*_) and for VOPc (*oxygen-up*) are shown in Figure 3. Figure 3c presents their unfolded acoustic and optical magnon bands, as well as the magnon spectrum of pristine CrSBr. We observe how the presence of the molecule shifts the energy of the magnons along the different directions of the k-path, the most notable effect being the increase in optical magnon energy induced by the [CpTi­(cot)] qubit. Figure 3d–g shows the calculated magnon spectra without unfolding. CrSBr-qubit heterostructures contain an additional low-energy band composed of qubit spins hybridized with acoustic magnons. It remains relatively flat due to the lack of direct qubit–qubit exchange, as qubits interact only via magnons in CrSBr. However, anticrossing occurs between this ″qubit band″ and the acoustic magnons of the 2D magnet, leading to strong hybridization of magnons and spin qubits as can be seen from the gaps open at anticrossing points. The weak indirect qubit–qubit interaction suggests that, in realistic scenarios with randomly distributed qubits, their spins may become localized. Nevertheless, qubit-magnon hybridization persists, manifesting as enhanced magnon scattering and magnon absorption by the qubits.

While a qubit layer modifies the magnon spectrum, individual spin qubits act as magnon emitters (Figure 4a). The qubit-magnon interaction is significantly stronger than qubit-phonon coupling, which typically results in qubit relaxation times on the order of 1 μs or larger^55–58^ for isolated molecules and larger than 100 ns for molecules on the substrate. Consequently, instead of decaying via incoherent spin-phonon processes, which dissipate quantum information, the excited qubit state relaxes through the emission of a magnon in a quantum-coherent state. Using our spin Hamiltonian framework, we analyze qubit-magnon interactions and find that qubit relaxation is characterized by relaxation times of 15.7 ps, 82.3 ps, and 7.19 ns for the *standing*_*cot*_, *standing*_*Cp*_, and *lying* orientations of [CpTi­(cot)], respectively, and 2.46 ns for VOPc (*oxygen-up*). Relaxation curves for various qubits and orientations are shown in Figure 4b. In Figures 4c-j, one can observe that qubits relax emitting a single magnon pulse with full spatial coherence, represented by magnon occupation probabilities *P*_*mag*_. We perform these simulations for *standing*_*cot*_ and *standing*_*Cp*_ orientations at different postrelaxation times, as well as for the less stable cases, i.e. *lying* [CpTi­(cot)] and *oxygen-down* VOPc (Figure S23). Initially, while the qubit remains significantly excited, the magnon state appears as a localized cloud around the qubit. Over time, a ring-like structure emerges, expanding with the magnon group velocity. In the *lying* orientation of the [CpTi­(cot)] qubit and for VOPc, the lower energies lead to magnon evolution on the nanosecond time scale and micrometer length scale. The shape of the coherent magnon state depends on the qubit relaxation time and the group velocity of magnons matching the qubit energy. One can observe that stronger exchange interaction *J*_*4*_ results in faster qubit relaxation, smaller, more rapidly forming ring with a thinner structure, and faster propagation of the signal (see also the energy dependence of group velocity discussed in section 6.5 in Supporting Information).

**Figure 4:**
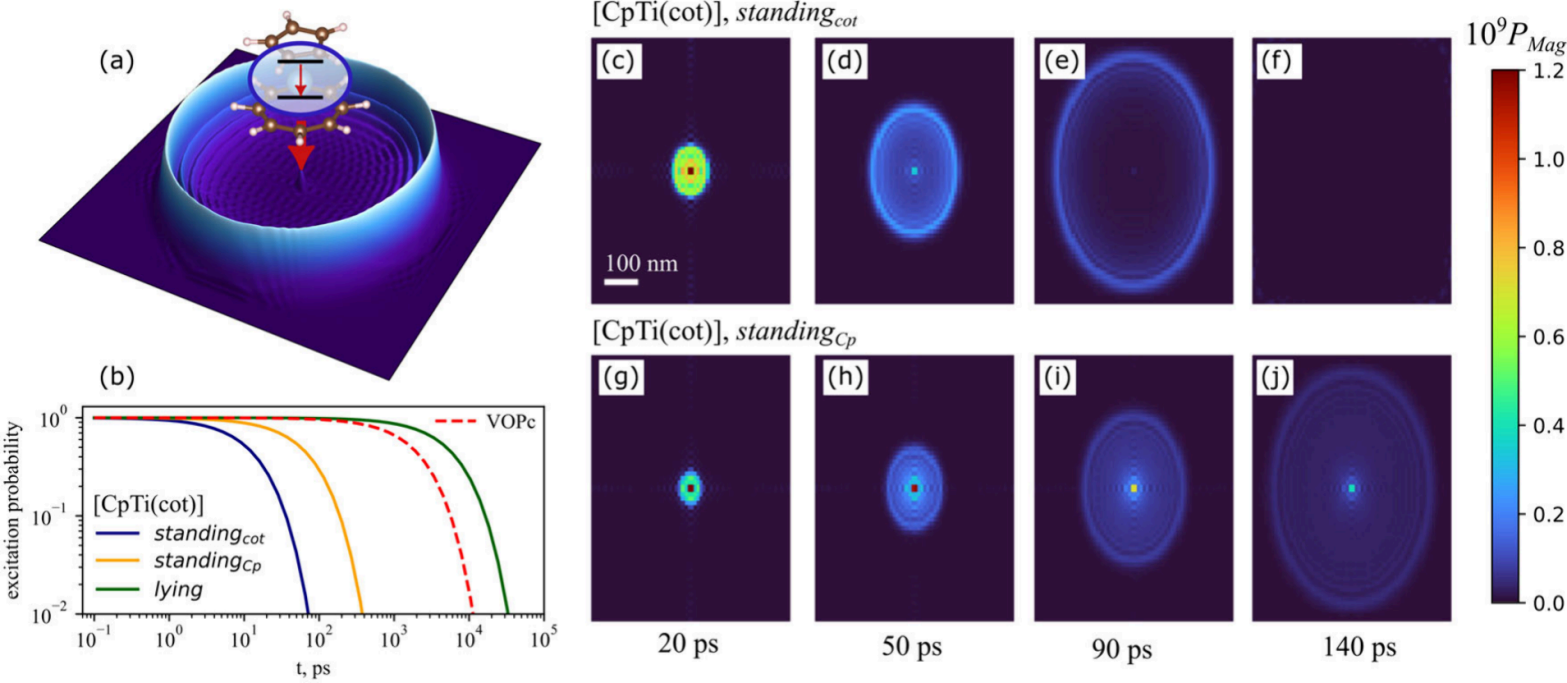
Molecular spin qubit relaxation. (a) Representation of the spin qubit relaxing by emitting a single-magnon pulse, (b) the time-dependent excitation probability of different qubits. (c-f) Calculated shapes of the single magnon pulse emitted by [CpTi­(cot)] qubit in *standing*_*cot*_ orientation at different postrelaxation times. The colors correspond to the time-dependent magnon occupation probability *P*_*mag*_. (g-j) Calculated shapes of the single magnon pulse emitted by [CpTi­(cot)] qubit in *standing*_*Cp*_ orientation.

The fast magnon dynamics is a core asset for any quantum protocol involving qubits and propagating fields, enabling high-speed quantum state preparation and processing. Furthermore, this is a core requisite for magnons to be useful in hybrid quantum platforms, as their high losses (compared to, e.g., photons) demand ultrafast protocols to allow using the unconventional magnon properties at minimized quantum coherence loss. At the same time, ultrafast protocols demand rapid magnon propagation, constrained by technological limits on device miniaturization. In this context, molecular qubits coupled to vdW magnetic materials with relatively strong exchange interactions offer a clear advantage—particularly when compared to systems like NV centers coupled via dipole–dipole interactions to YIG. These exchange-based couplings not only accelerate the generation of magnonic states but also enhance their propagation speed. This strategy may prove crucial in overcoming the decoherence challenge in quantum magnonics — a critical step toward effectively integrating magnons into quantum platforms (e.g., as transducers between optical, mechanical, and/or microwave degrees of freedom, or as fast local nodes to perform nonlinear operations on states of – otherwise very linear – microwave electromagnetic modes).

An optimal way of increasing the molecule–substrate exchange coupling may be the introduction of an alkyl group in any of the 8-member or 5-member ring of [CpTi­(cot)], as this will enhance the donor character of these rings. In VOPc, weak coupling arises from an unpaired electron in the *d*_*xy*_ orbital, which lacks strong overlap with the substrate and bonded atoms. This results from the strong crystal field of the V=O bond, which lifts orbital degeneracy. Selecting qubits with weaker axial ligand fields could enhance molecule–substrate coupling. Furthermore, to strengthen molecule–substrate coupling, qubits with S = 1/2 organic radicals like TEMPO (2,2,6,6-Tetramethylpiperidine-1-oxyl) and nitronylnitroxide are promising candidates, as they can be engineered for chemisorption onto the substrate through appropriate chemical modifications.^24,59–61^ Additionally, Br vacancies in CrSBr, which are positively charged, could facilitate electron donation from the molecule due to the electron deficiency in the substrate.

In summary, we investigate the electronic structure, magnetic properties and quantum magnon dynamics of [CpTi­(cot)] and VOPc spin qubits deposited on single-layer CrSBr. Besides tuning the magnon band structure of the 2D magnet, our calculations predict that qubits relax emitting an ultrafast single magnon pulse with full spatial coherence. We observe the most pronounced effects in the hybrid heterostructure formed by [CpTi­(cot)] on CrSBr, in which we demonstrate that the relaxation time can be selectively tuned from 16 ps to 7 ns as a function of molecular orientation. Additionally, we provide a detailed microscopic understanding of the qubit-substrate coupling, shedding light on the rational exploitation of coherent magnon dynamics in hybrid molecular/2D magnetic heterostructures. This work paves the way to hybrid quantum platforms that harness magnons in an ultrafast regime, thus leveraging their unconventional properties while minimizing decoherence caused by magnon loss.

## Supplementary Material



## Notes

Computational methods; electronic structure of qubits in the gas phase; electronic structure of [CpTi­(cot)]@CrSBr and VOPc@CrSBr heterostructures; magnetic exchange of the heterostructures; dipole–dipole energy; spin dynamics of the heterostructures (PDF)
