# Supplementary material for: Coupling Molecular Spin Qubits with 2D Magnets for Coherent Magnon Manipulation
Source: Nano Lett. 2025 Jun 17;25(26):10457–64. doi: 10.1021/acs.nanolett.5c01937 (PMC12232374; doi:10.1021/acs.nanolett.5c01937)
Supplement: Supplementary file 1 [file nl5c01937_si_001.pdf]

Supporting Information

Coupling Molecular Spin Qubits with 2D Magnets for Coherent Magnon Manipulation

Sourav Dey[†1,3], *Gonzalo Rivero-Carracedo*[†1], *Andrei Shumilin*[1], *Carlos Gonzalez-Ballestero*[2]* *and José J. Baldoví*[1]*

[1]Instituto de Ciencia Molecular (ICMol), Universitat de Valencia, c/Catedrático José Beltrán, 2. Paterna 46980, Spain

[2]Institute for Theoretical Physics, Vienna University of Technology (TU Wien), Wiedner Haupstraße 8-10, 1040 Vienna, Austria

[3] Washington State University, Pullman, WA 99164, USA

Email: j.jaime.baldovi@uv.es, carlos.gonzalez-ballestero@tuwien.ac.at

1. Computational details 3

2. Electronic structure of molecular spin qubits in gas phase 4

3. Electronic structure of [CpTi(cot)]@CrSBr and VOPc@CrSBr heterostructures 7

3.1. Orientation of molecules on the surface, structural parameters and adsorption energy 7

3.2. Charge and spin density distributions. 9

4. Magnetic exchange of [CpTi(cot)]@CrSBr and VOPc@CrSBr heterostructures 17

4.1) $standing_{COT}$ 18

4.2) $standing_{Cp}$ 19

4.3) *lying* 19

4.4) *oxygen-up* 20

4.5) *oxygen-down* 20

5. Calculation of the dipole-dipole interaction energy E_{dd} 22

6. Spin dynamics of qubit in [CpTi(cot)]@CrSBr and VOPc@CrSBr heterostructures 22

6.1. Unfolding magnon bands. 22

6.2. Qubit relaxation: theory. 23

6.3. Qubit Relaxation: Justification of the Markov Approximation 26

6.4. Qubit relaxation of [CpTi(cot)]@CrSBr and VOPc@CrSBr heterostructures in less favorable configurations. 27

6.5. Magnon group velocities 28

7. References 30

1. Computational details

The gas phase geometry optimization of the qubit molecules was performed with density-functional theory (DFT) calculations with the ORCA (v. 5.0.3) package to understand their electronic structure (Figures S1-S2).[1] The DFT calculations were performed with PBE0 functional and def2- TZVP basis set.[2] The frontier orbitals were calculated with the def2/J auxiliary basis set and RIJCOSX approximation.[3]

All the spin-polarized DFT calculations on the CrSBr monolayer were carried out using the Quantum ESPRESSO package.[4] The Perdew–Burke–Ernzerhof (PBE) exchange-correlation functional was employed in the framework of generalised gradient approximation (GGA).[5] The lattice parameters and atomic coordinates were fully relaxed using Broyden–Fletcher–Goldfarb–Shanno (BFGS)[6] algorithm within the convergence criteria of 1×10^{-3} Ry/au forces for each atom and 1×10^{-4} Ry energy difference between two consecutive relaxation steps. We have used 60 and 600 Ry for kinetic energy and charge density cutoffs for the expansion of the electronic wavefunction. All the pseudopotentials were taken from the solid-state ultrasoft library of the Quantum ESPRESSO package. To avoid unphysical interlayer interactions along c directions, an 18 Å vacuum spacing was employed. A G-centered 8 × 8 × 1 k-point Monkhorst–Pack grid was used to integrate the Brillouin zone.

Thereafter, we have constructed 4 × 4 and 6 × 6 supercells for the [CpTi(cot)]@CrSBr and VOPc@CrSBr heterostructures to ensure a minimum distance of 8 Å between the neighbouring molecules as we are interested in investigating the individual effect of qubits on the magnetic properties of CrSBr. We performed full optimization of the atomic coordinates, while keeping the optimized lattice parameters of CrSBr, as the effect of a single molecule on the lattice parameter of the substrate is expected to be negligible.

To consider the vdW interactions between qubit and the substrate we have employed Grimme-D2 dispersion corrections in our calculations.[7] The electron correlations in 3d orbitals of Cr and V, have been taken into account using an U_{eff} =3eV (U=4eV and J_H =1eV) using Liechtenstein's formulation for both of them.[8] The Brillouin zone was sampled by Γ-centred 4×4×1 (2×2×1) k-point grid for [CpTi(cot)]@CrSBr (VOPc@CrSBr). We have calculated the adsorption energy as follows,

$$E_{ads} = E_{CrSBr+qubit} - E_{CrSBr} - E_{qubit} \quad (1)$$

where $E_{CrSBr+qubit}$, E_{CrSBr}, E_{qubit} correspond to the energies of the relaxed geometry of hybrid heterostructure, pristine CrSBr, and isolated qubit, respectively.

The charge transfer between the qubit and CrSBr was calculated using Bader charge population analysis.[9] To visualize the charge density difference (CDD), the charge density of isolated qubits and pristine CrSBr monolayers were subtracted from the total charge density of the hybrid heterostructure using the following formula,

$$\Delta\rho = \rho_{CrSBr+qubit} - \rho_{CrSBr} - \rho_{qubit} \quad (2)$$

where, $\rho_{CrSBr+qubit}$ and ρ_{CrSBr} corresponds to the total electron densities of monolayer CrSBr with and without qubit respectively and ρ_{qubit} denotes the electron density of the isolated qubit. It is worth mentioning that separate qubit and CrSBr monolayer should have the same distorted geometry as in the hybrid heterostructure.

The magnetic exchange interactions (J_1, J_2 and J_3) in the CrSBr monolayer, along with CrSBr — qubit exchange interaction J_4, are determined by mapping the total energies of five distinct magnetic configurations, one ferromagnetic and four antiferromagnetic, onto a classical Heisenberg Hamiltonian.

Magnon spectra are computed using the linear approximation of the Holstein–Primakoff boson expansion,[10] with calculations performed using the RAD-tools software.[11] The unfolding procedure is carried out by analyzing magnon wavefunctions, as detailed **section 6.1**. Magnon relaxation rates and probability distributions are obtained within the Weisskopf-Wigner framework[48], justified by the efficient magnon transport in CrSBr, which ensures Markovian qubit relaxation. The corresponding quantum-mechanical calculations are provided in **sections 6.2** and **6.3**.

2. Electronic structure of molecular spin qubits in gas phase

First, we performed DFT calculations on isolated [CpTi(cot)] and VOPc to understand their electronic structure. The calculations on [CpTi(cot)] reveal the splitting of the d orbitals into non-bonding, bonding and anti-bonding sets. The interaction of the 3d orbital of the metal with the p electron clouds of the rings leads to the latter two sets. The non-bonding d_{z^2} orbital is found to be the singly occupied molecular orbital (SOMO), which acts as magnetic orbital. The d_{xy} and $d_{x^2-y^2}$ orbitals of Ti atom selectively interact with the COT^{2-} (cyclooctatetraene) ring, which leads to the formation of a δ-bond. Besides, the d_{xz} and d_{yz} orbitals undergo p-bond formation with the Cp (cyclopentadienyl) ring. As shown in Figure S1, two bonding and two antibonding combinations were yielded from these interactions, which are found to be based on ligand and metal, respectively. In the case of VOPc, the calculations reveal that the unpaired electron resides in the d_{xy} orbital, which acts as a magnetic orbital. The d_{z^2} orbital of vanadium is found to form a σ-bond with the 2p orbital of oxygen, while the d_{xz} and d_{yz} form p-bond with the latter. The interactions of the $d_{x^2-y^2}$ orbital of vanadium with the 2p orbitals of nitrogen lead to the formation of a s-bond between them. We have shown the bonding and antibonding combinations resulting from this interaction in Figure S2. The bonding and antibonding orbitals in VOPc are found to be higher in energy compared to [CpTi(cot)] as σ and π bonds are stronger compared to the δ and π bonds in the latter. Previous bonding analyses of similar lanthanoarene complexes have revealed fluxional hapticity, suggesting that an Atoms in Molecules (AIM) analysis would be valuable for a deeper understanding of their electronic structure.[12,13]

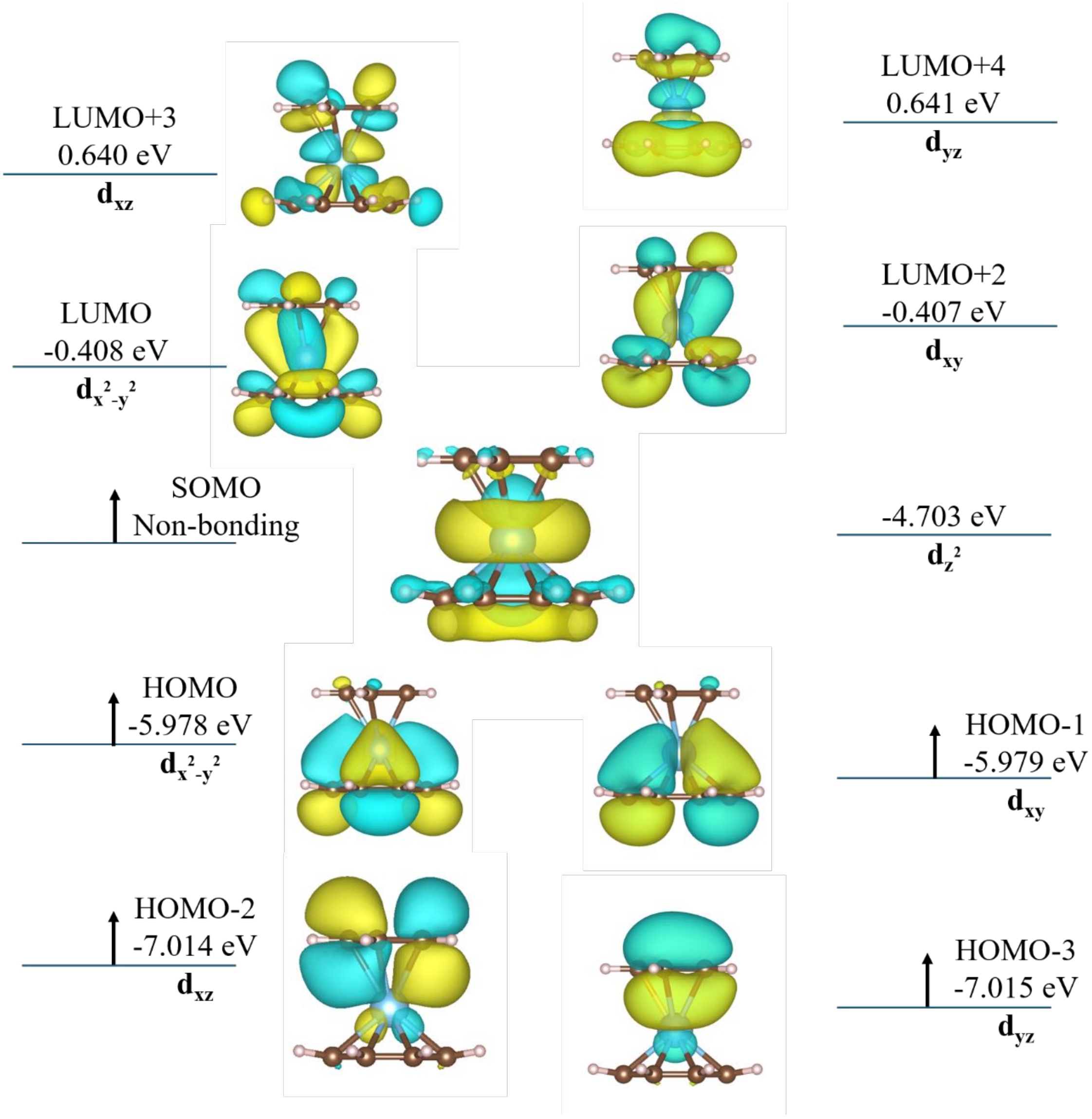

Figure S1: Energies and contour surfaces of frontier molecular orbitals of [CpTi(cot)] (spin up). The isovalue is set to 0.012 eV·Å^{-3}.

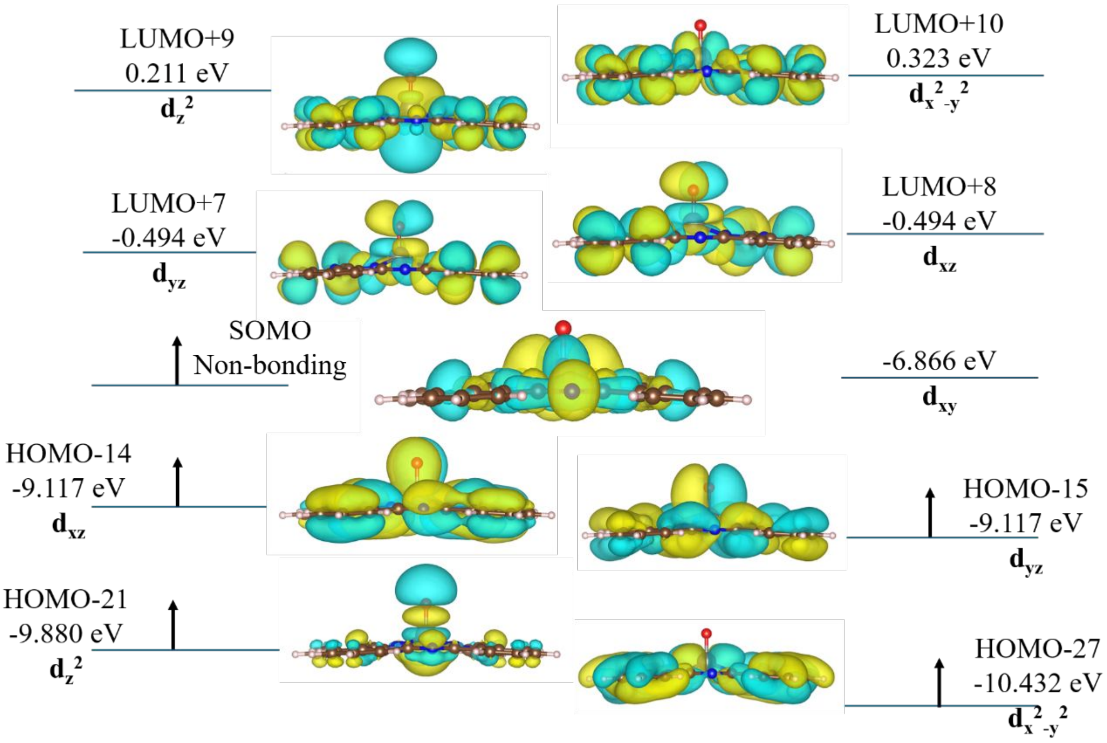

Figure S2: Energies and contour surfaces of frontier molecular orbitals of VOPc (spin up). The isovalue is set to 0.012 eV·Å^{-3}.

3. Electronic structure of [CpTi(cot)]@CrSBr and VOPc@CrSBr heterostructures

3.1. Orientation of molecules on the surface, structural parameters and adsorption energy

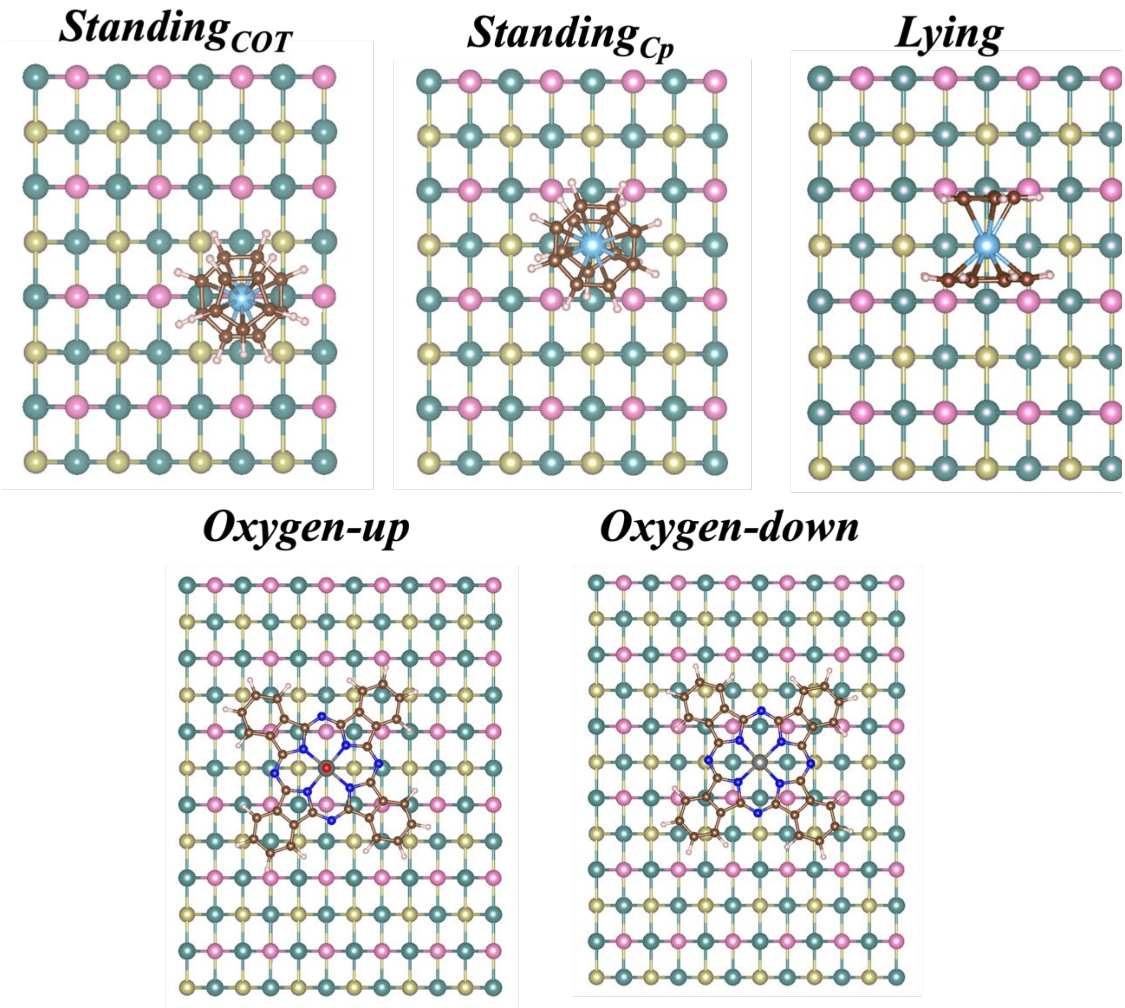

Figure S3: (Top) Top views of optimized adsorption geometries of [CpTi(cot)] on the most stable site of CrSBr. (Down) Top views of optimized adsorption geometries of VOPc on the most stable site of CrSBr.

Table S1: The bonding and non-bonding distance (Å) in [CpTi(cot)]@CrSBr heterostructure (see Figure S4 for the lables).

Distance	Only molecule	*Standing*$_{COT}$	*Standing*$_{Cp}$	*Lying*
Z_1	2.028	1.972	1.974	1.976
Z_2	1.440	1.348	1.380	1.368
Z_3		2.754	3.090	2.960
Z_4		4.191	5.243	5.914

Table S2: The bonding and metal-substrate distance (Å) in VOPc@CrSBr heterostructure.

Distance	Only molecule	*Oxygen-up*	*Oxygen-down*
V-O	1.580	1.590	1.590
V-N	2.026	2.032	2.036
Out of plane distance of V	0.575	0.547	0.562
Molecule-substrate (like Z_3 in Figure S4)		2.950	2.994

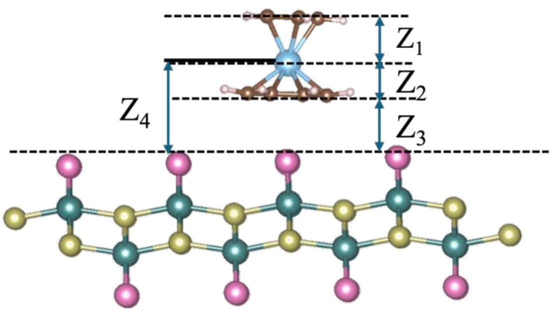

Figure S4: A schematic model for bonding distance in CpTicot and molecule-substrate and metal-substrate distance in [CpTi(cot)]@CrSBr heterostructure.

Table S3: The adsorption energy values (eV) of [CpTi(cot)] for the four adsorption sites of CrSBr. The values in bold indicate the preferential site for adsorption.

Site	*Standing$_{COT}$*	*Standing$_{Cp}$*	*Lying*
Br-top	**-0.93**	-0.63	-0.52
Cr-top	-0.79	-0.63	-0.52
S-top	-0.79	**-0.67**	**-0.53**
Hollow-top	-0.76	-0.64	-0.52

Table S4: The adsorption energy values (eV) of VOPc for the four adsorption sites of CrSBr. The values in bold indicate the preferential site for adsorption.

Site	*Oxygen-up*	*Oxygen-down*
Br-top	-1.59	-0.54
Cr-top	**-1.64**	-0.58
S-top	-1.60	**-0.58**
Hollow-top	-1.59	-0.58

3.2. Charge and spin density distributions.

Following the same methodology as in our previous works based on molecular/CrSBr hybrid heterostructures,[14,15] we calculated charge transfer between qubits and CrSBr and the modification of spin density after the deposition. Our calculations evidence the transfer of electrons from the molecule to the substrate for all molecular orientations and adsorption sites. For [CpTi(cot)], the largest charge transfer (0.46e) is estimated for standingcot orientation (which presents the largest adsorption energy), followed by 0.37e for both standingCp and lying orientations. The charge density difference (CDD) plot suggests charge depletion (blue) in the Cp and cot rings, whereas the charge accumulation (yellow) occurs in the Br atoms of the substrate due to the high electronegativity of the latter (see Figures S5 (a,b) and S6). In the case of VOPc, a charge transfer of 0.24e and 0.16e is calculated for oxygen-up and oxygen-down orientations, respectively. Here, charge depletion occurs from the phthalocyanine macrocycle (Figures S5 (d,e) and S7). Then, to understand the effect of electron delocalization on the magnetic properties, we calculate the spin density of the heterostructure and compare it with the isolated molecule (Figures S5 (c,f) and S8).

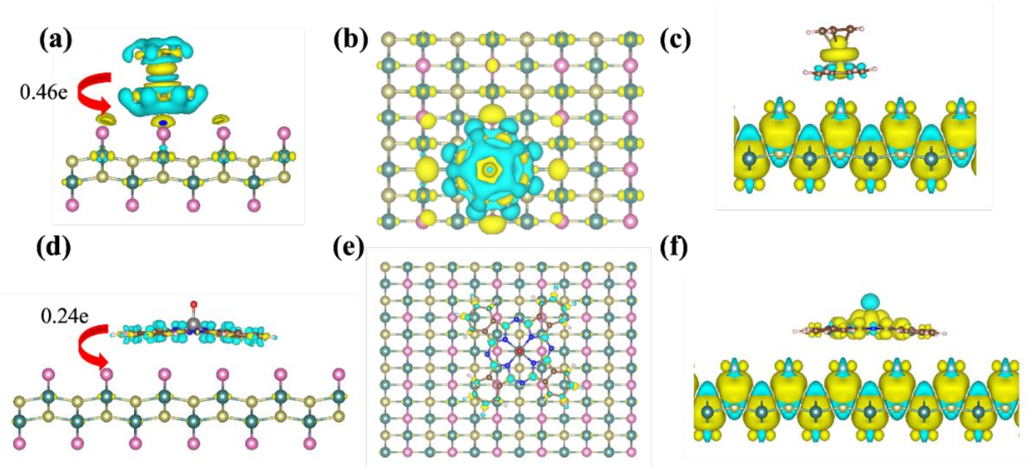

Figure S5: Charge density difference (CDD) plots of the CrSBr heterostructure for the *standing*$_{cot}$ orientation of [CpTi(cot)]: (a) side view and (b) top view, and for the *oxygen-up* orientation of VOPc: (d) side view and (e) top view. Yellow and blue regions indicate charge accumulation and depletion, respectively, with an isosurface value of 0.0005 eVÅ^{-3}. Spin density distribution in the CrSBr heterostructure for (c) the *standing*$_{cot}$ orientation of [CpTi(cot)] and (f) the *oxygen-up* orientation of VOPc, with an isovalue of 0.001 eVÅ^{-3}.

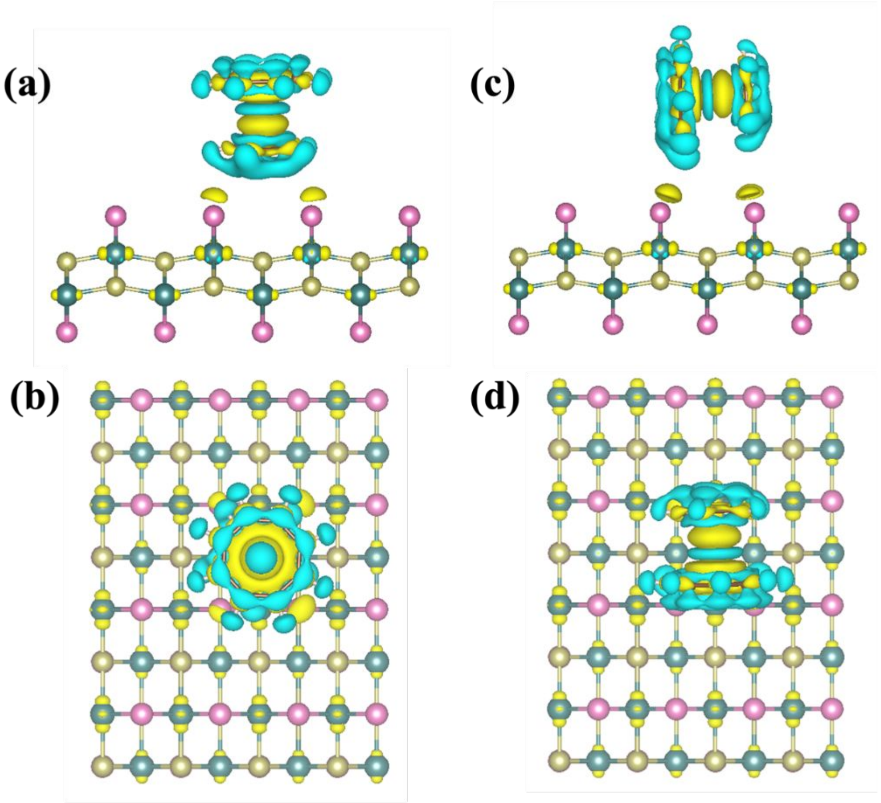

Figure S6: The charge density difference (CDD) plots of the CrSBr heterostructure of (a) *standing*$_{Cp}$ orientation of [CpTi(cot)] (side view) (b) *standing*$_{Cp}$ orientation of CpTicot (top view) (c) *lying* orientation of [CpTi(cot)] (side view) (d) *lying* orientation of [CpTi(cot)] (top view). The yellow and blue colour represents the region with charge accumulation and depletion respectively. The isosurface value was set to 0.0005 eVÅ^{-3}.

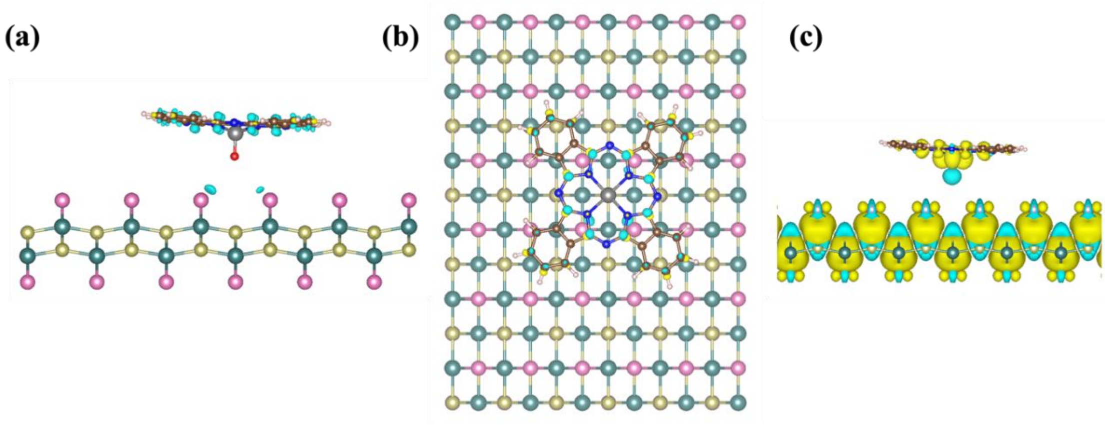

Figure S7: The CDD plots for the VOPc@CrSBr heterostructure in the *oxygen-down* orientation (a) side view (b) top view. The isosurface value was set to 0.0005 eVÅ^{-3}. (c) Spin density distribution with the isovalue set to 0.001 eVÅ^{-3}.

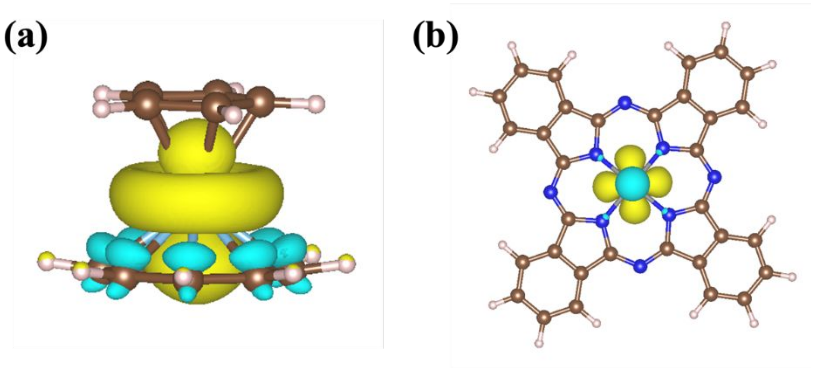

Figure S8: The spin density distribution of (a) [CpTi(cot)] (b) VOPc. The isovalue is set to 0.001 eVÅ^{-3}.

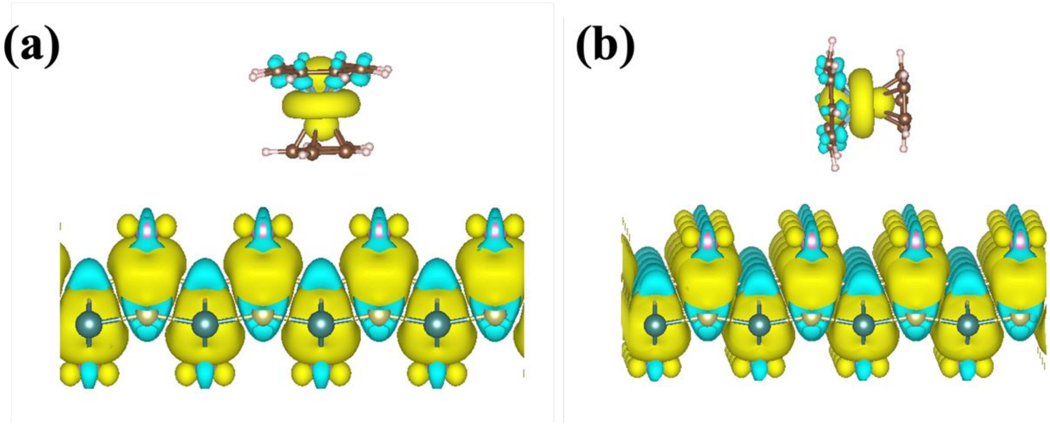

Figure S9: The spin density distribution of the CrSBr heterostructure of (a) $standing_{Cp}$ orientation of [CpTi(cot)] (b) *lying* orientation of [CpTi(cot)]. The isovalue is set to 0.001 eVÅ^{-3}.

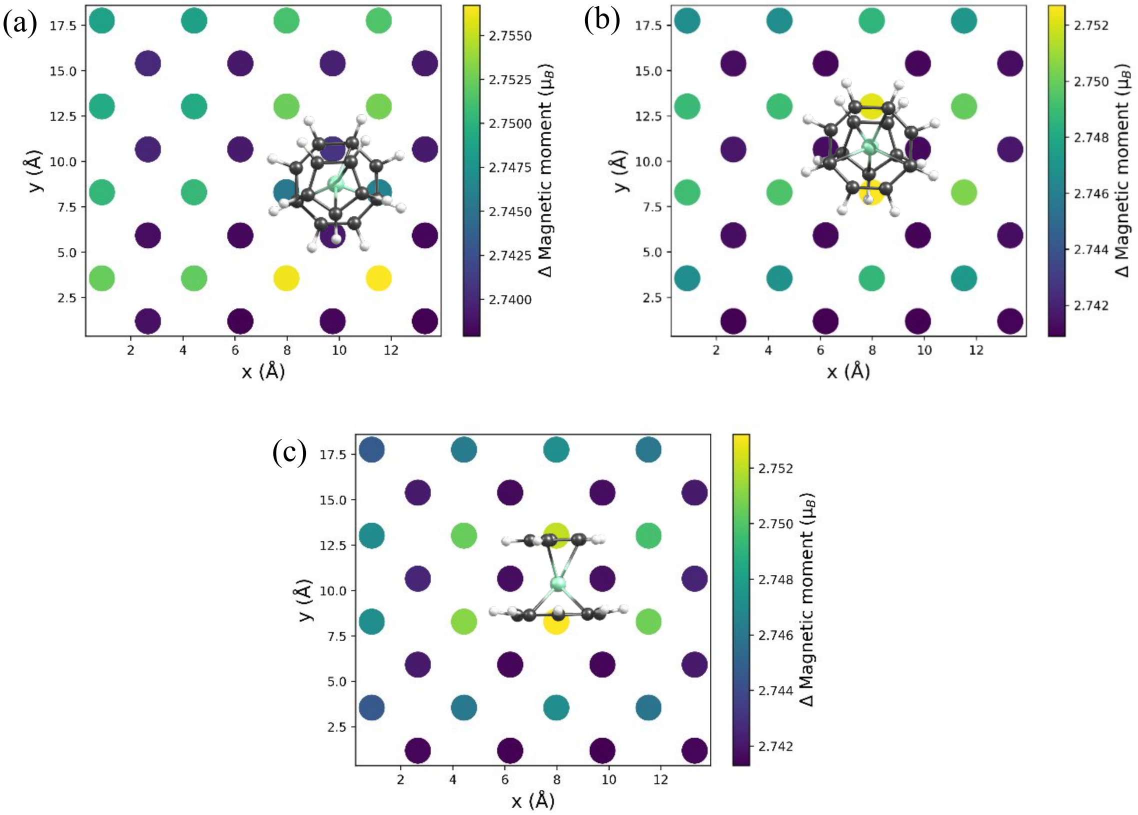

Figure S10. Schematic top view of the [CpTi(cot)] molecule deposited in the substrate in the (a) *standing*$_{cot}$, (b) *standing*$_{Cp}$, and (c) *lying* configurations. The Cr atoms of the surface are depicted as colored balls, where the different colors show the change in their magnetic moments.

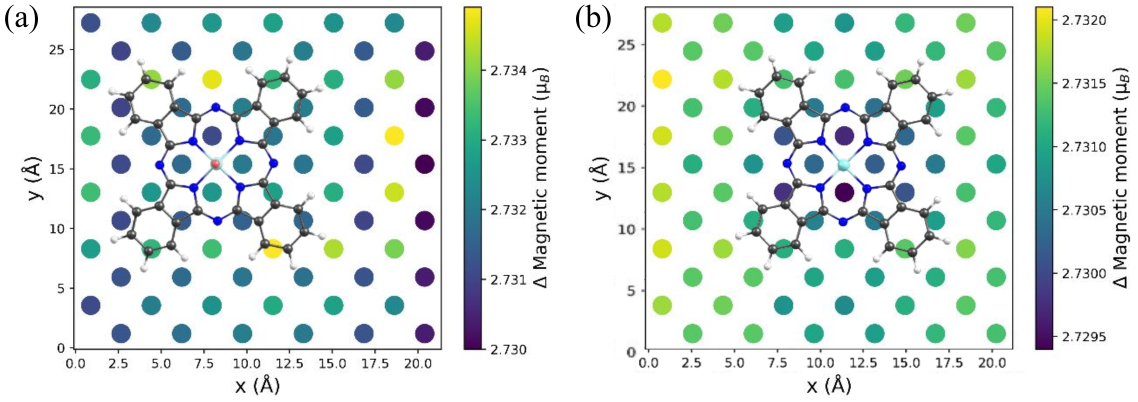

Figure S11. Schematic top view of the VOPc molecule deposited in the substrate in the (a) *oxygen-up*, and (b) *oxygen-down* configurations. The Cr atoms of the surface are depicted as colored balls, where the different colors show the change in their magnetic moments.

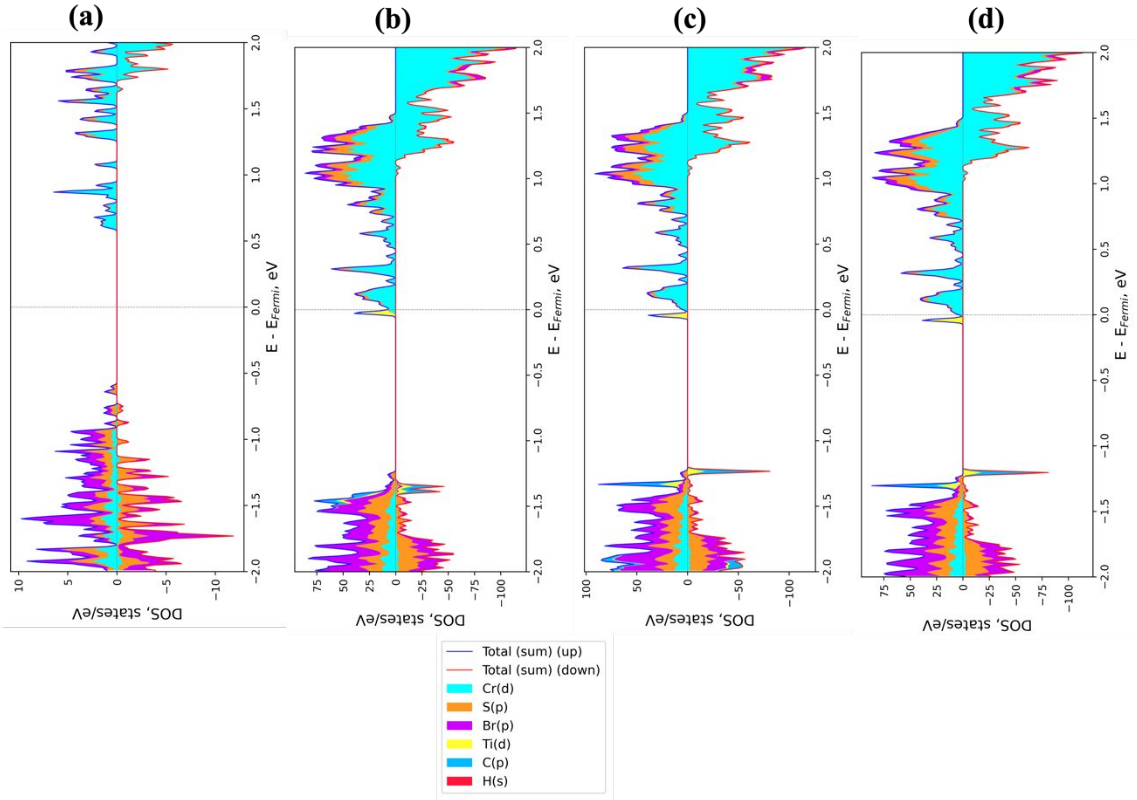

Figure S12: The calculated projected density of states of (a) pristine CrSBr and heterostructure of [CpTi(cot)] with CrSBr in (b) *standing*$_{cot}$ (c) *standing*$_{Cp}$ and (d) *lying* orientations.

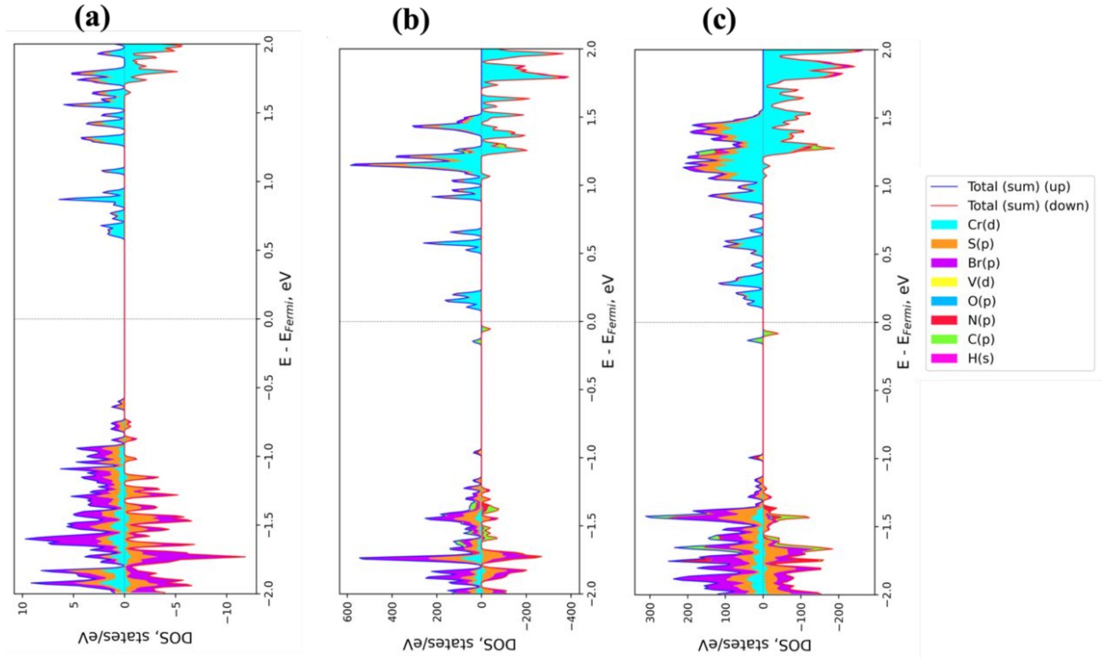

Figure S13: The calculated projected density of states of (a) pristine CrSBr and heterostructure of VOPc with CrSBr in (b) *oxygen-up* (c) *oxygen-down* orientations.

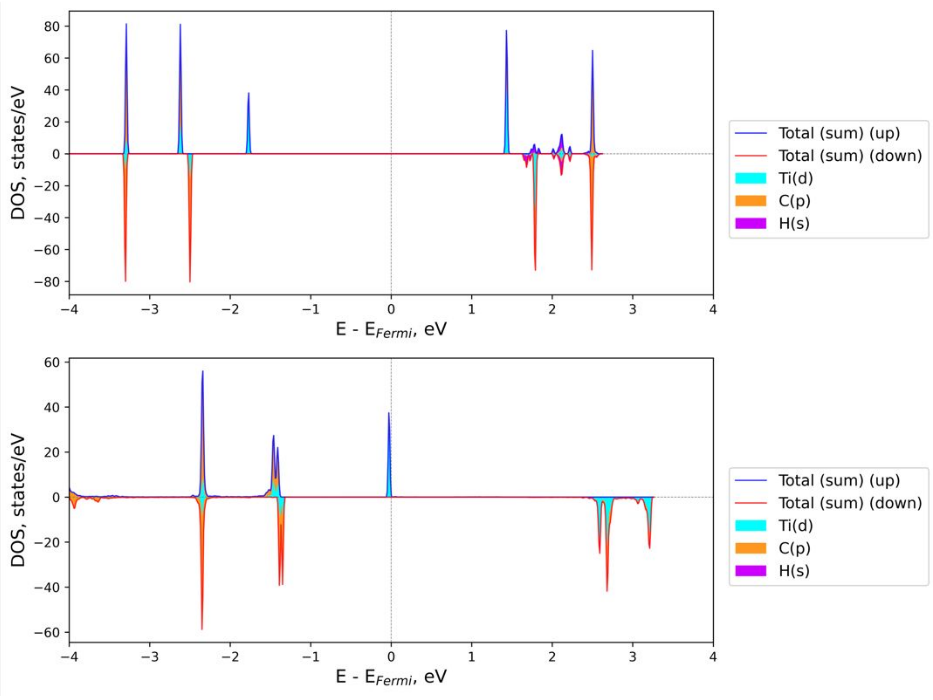

Figure S14: The calculate pdos of free (top) and adsorbed-distorted (*standing*$_{COT}$) [CpTi(cot)] molecule.

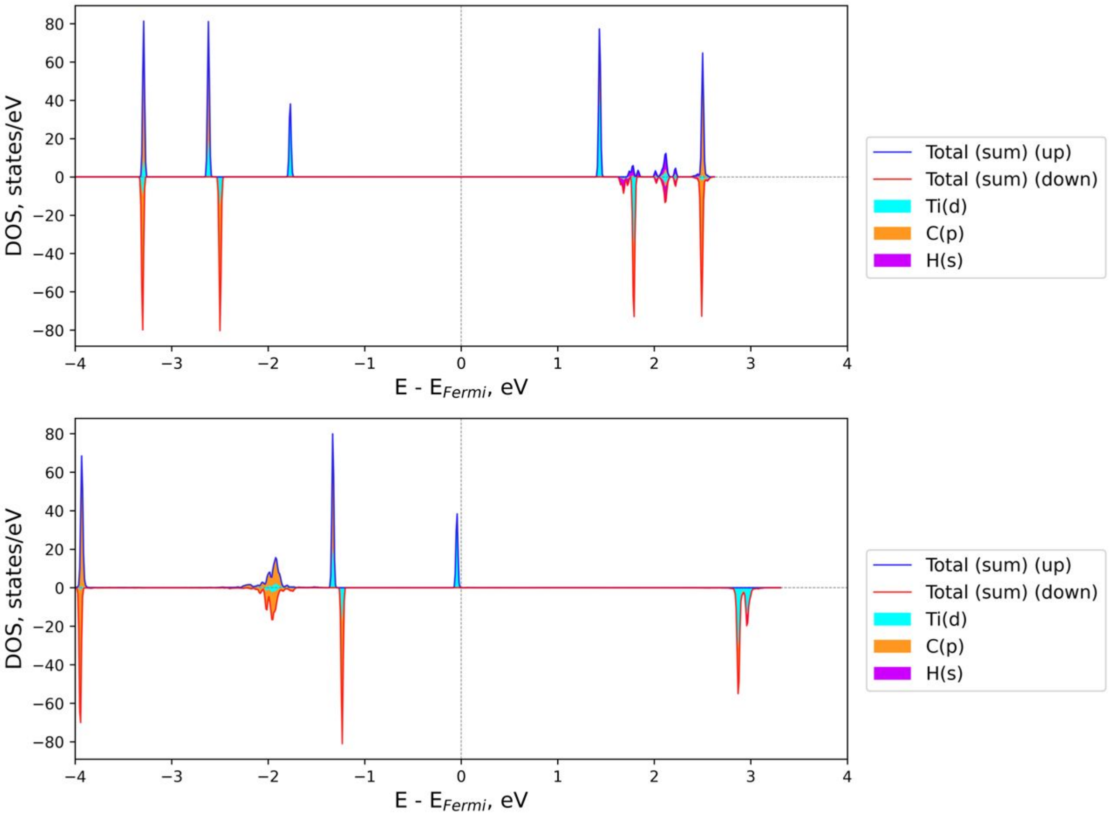

Figure S15: The calculate pdos of free (top) and adsorbed-distorted (*standing*$_{Cp}$) of [CpTi(cot)] molecule.

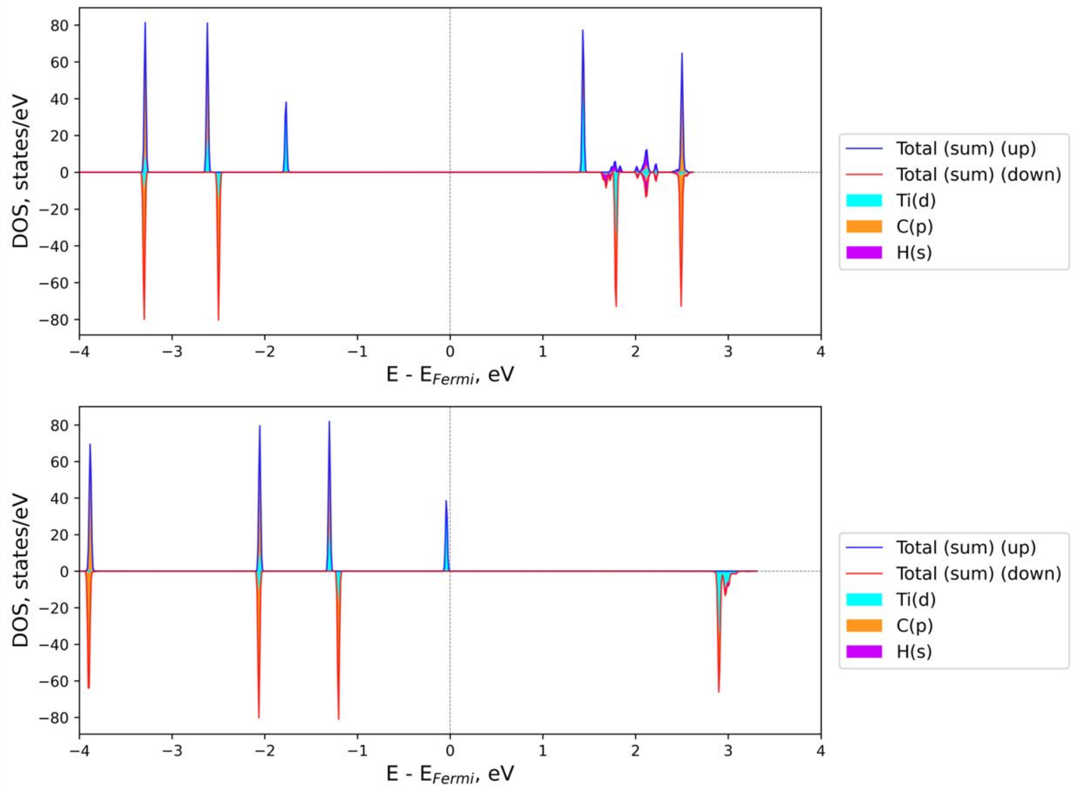

Figure S16: The calculate pdos of free (top) and adsorbed-distorted (*lying*) of [CpTi(cot)] molecule.

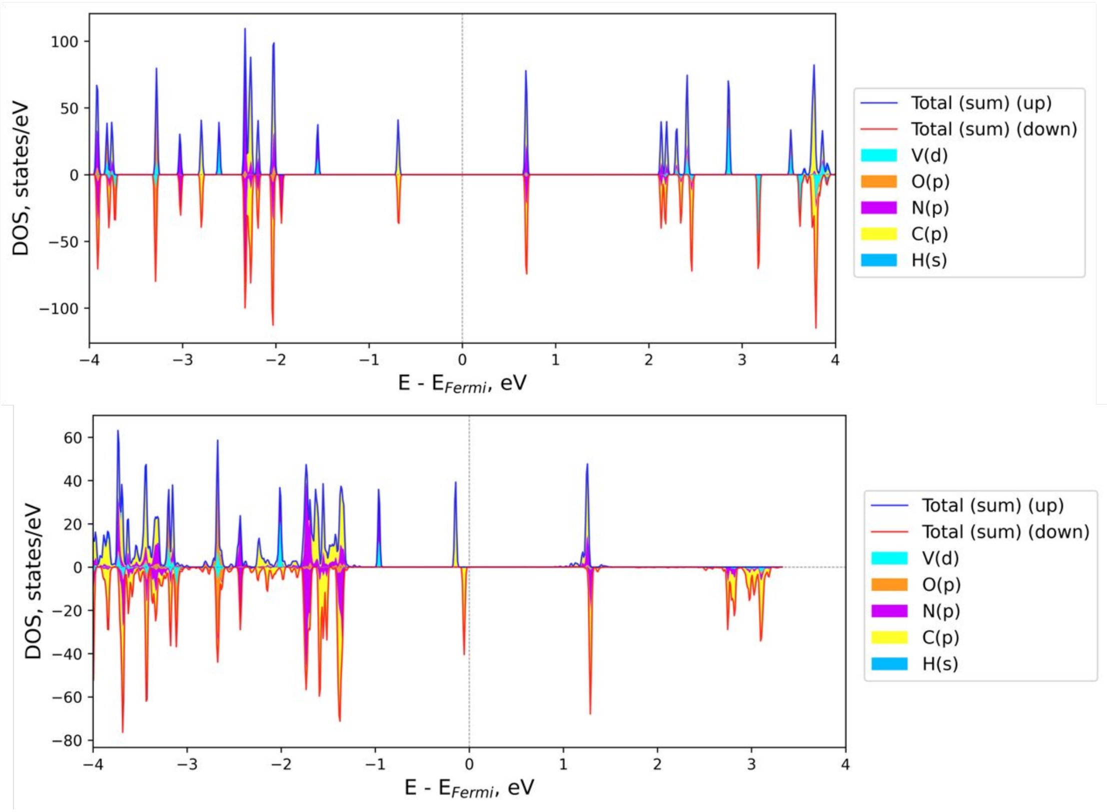

Figure S17: The calculate pdos of free (top) and adsorbed-distorted (*oxygen-up*) VOPc molecule.

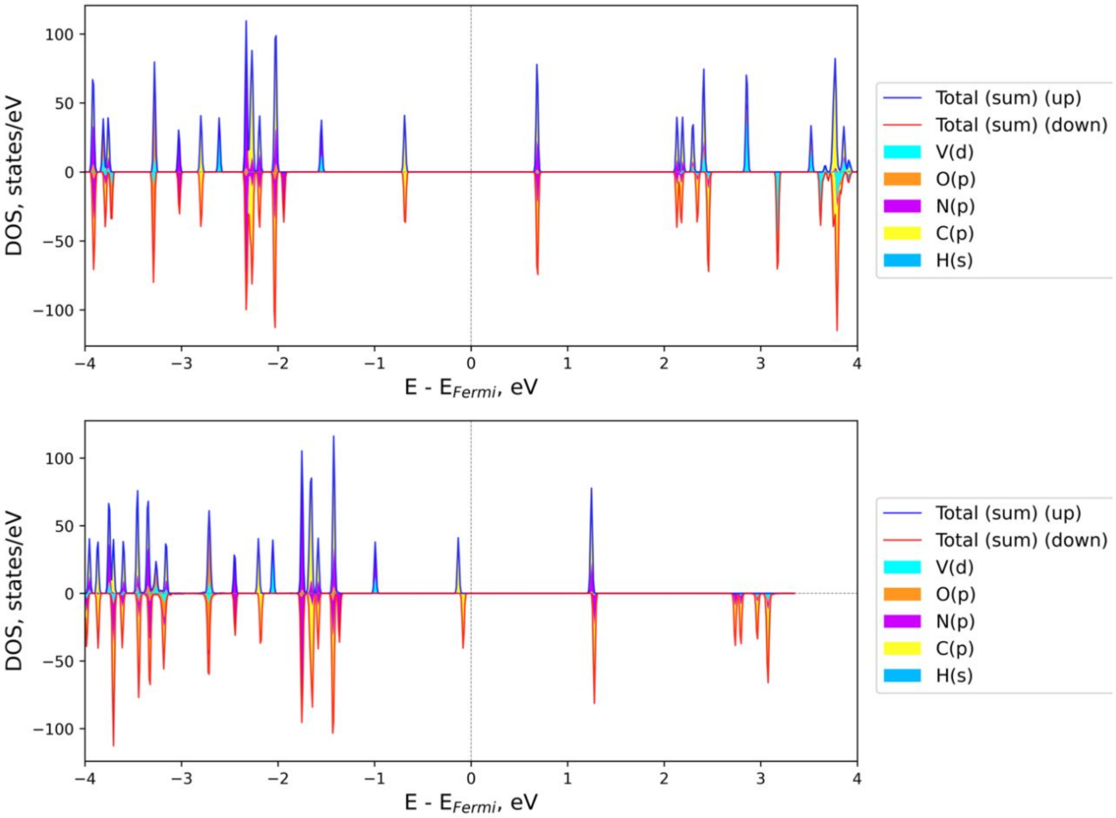

Figure S18: The calculated pdos of free (top) and adsorbed-distorted (*oxygen-down*) VOPc molecule.

4. Magnetic exchange of [CpTi(cot)]@CrSBr and VOPc@CrSBr heterostructures

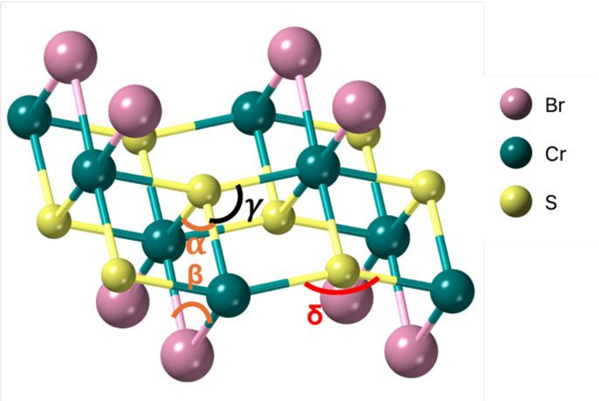

Figure S19: The characteristic angles of CrSBr.

Table S5: The values of characteristics angle (°) of pristine CrSBr and its heterostructure with [CpTi(cot)] and VOPc. Note that, we have considered the average angle in the hetrostructure.

Angle	CrSBr	[CpTi(cot)]@CrSBr			VOPc@CrSBr
		$standing_{cot}$	$standing_{Cp}$	*lying*	*oxygen-up*
α	95.8	96.0	96.0	96.0	96.0
β	89.8	88.9	89.0	89.0	89.1
γ	97.5	96.4	96.5	96.5	96.5
δ	157.6	160.8	160.6	160.6	160.5

For the calculation of the magnetic exchange parameters, namely J_1, J_2 and J_3 in the substrate and J_4 between substrate and molecule, we considered different magnetic configurations as depicted in Figure S19-20, and mapped their DFT calculated energies onto a Heisenberg Hamiltonian with the form:

$$\hat{H} = -\sum_{ij} J_{ij} \hat{s}_i \hat{s}_j$$

where double counting is present, and spins are normalized to 1.

(i) [CpTi(cot)]

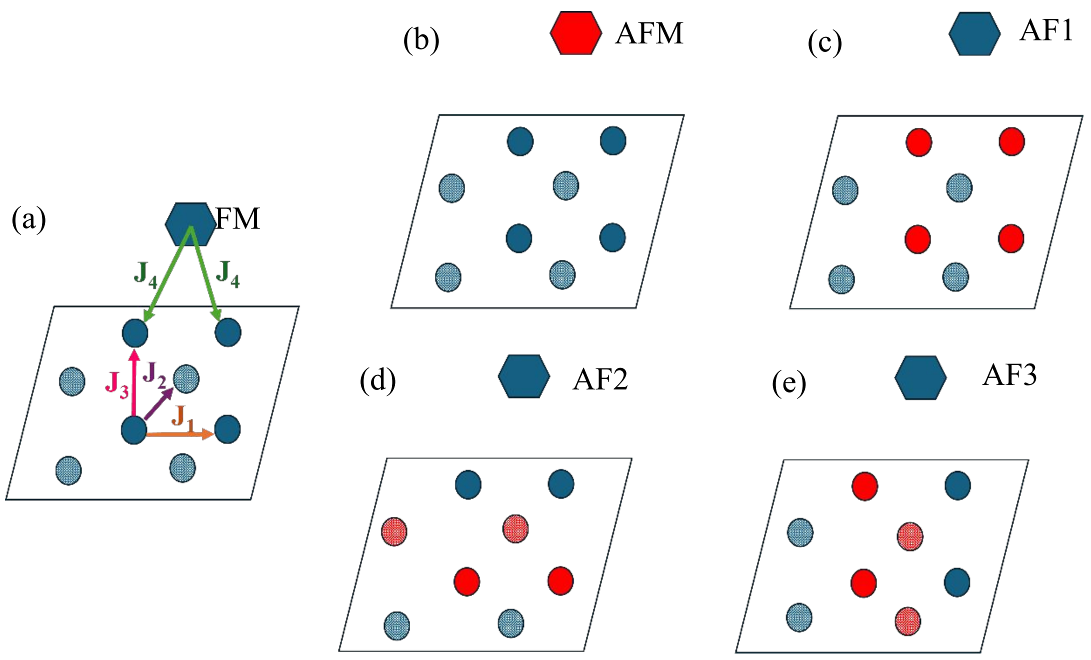

Figure S20: Schematic representation of the Cr magnetic atoms of the surface and the Ti atom of the [CpTi(cot)] molecule. We show the magnetic exchange parameters (a) and the different magnetic configurations considered in the calculations (a-e). Blue (red) represents spin up (down). Note that in this material we have two different z coordinates for Cr atoms, where the dotted balls represent the ones that are lower in height.

In the case of [CpTi(cot)], we consider that the molecule is magnetically coupled to 2 Cr atoms from the surface, which are the nearest neighbours. Considering positive (+) sign for ferromagnetic coupling and negative (-) sign for antiferromagnetic coupling we can obtain energy expressions for each magnetic configuration. Hence, by substracting the energy of the ferromagnetic configuration (E_{FM}) to each expression to get rid of the non-magnetic energetic term, we get the following set of equations for the different orientations of the [CpTi(cot)] molecule depending on their calculated most stable site:

4.1) *standing*$_{COT}$

$$E_{AFM} - E_{FM} = -4J_4 S_{Cr} S_{Ti}$$

$$E_{AF1} - E_{FM} = -64J_2 S_{Cr}^2 - 4J_4 S_{Cr} S_{Ti}$$

$$E_{AF2} - E_{FM} = (-32J_2 - 32J_3) S_{Cr}^2$$

$$E_{AF3} - E_{FM} = (-32J_1 - 32J_2) S_{Cr}^2 - 2J_4 S_{Cr} S_{Ti}$$

4.2) ***standing$_{Cp}$***

$$E_{AFM} - E_{FM} = -4J_4S_{Cr}S_{Ti}$$

$$E_{AF1} - E_{FM} = -64J_2S_{Cr}^2 - 4J_4S_{Cr}S_{Ti}$$

$$E_{AF2} - E_{FM} = (-32J_2 - 32J_3)S_{Cr}^2 - 2J_4S_{Cr}S_{Ti}$$

$$E_{AF3} - E_{FM} = (-32J_1 - 32J_2)S_{Cr}^2 - 4J_4S_{Cr}S_{Ti}$$

4.3) ***lying***

$$E_{AFM} - E_{FM} = -4J_4S_{Cr}S_{Ti}$$

$$E_{AF1} - E_{FM} = -64J_2S_{Cr}^2 - 4J_4S_{Cr}S_{Ti}$$

$$E_{AF2} - E_{FM} = (-32J_2 - 32J_3)S_{Cr}^2 - 2J_4S_{Cr}S_{Ti}$$

$$E_{AF3} - E_{FM} = (-32J_1 - 32J_2)S_{Cr}^2 - 4J_4S_{Cr}S_{Ti}$$

(ii) VOPc

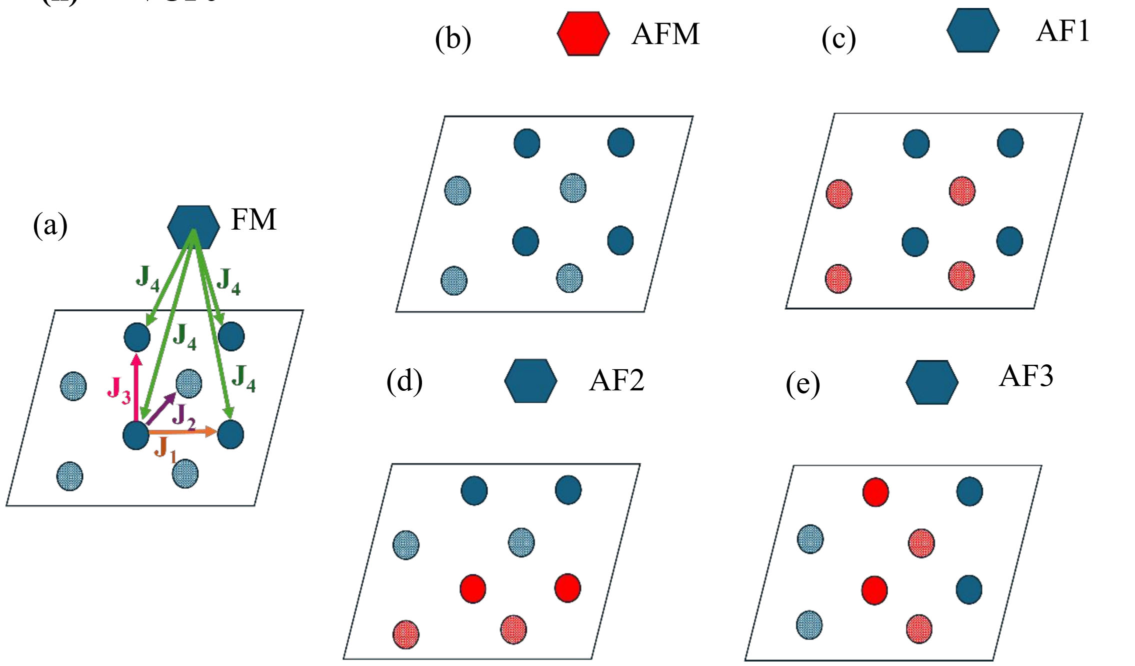

Figure S21. Schematic representation of the Cr magnetic atoms of the surface and the V atom of the VOPc molecule. We show the magnetic exchange parameters (a) and the different magnetic configurations considered in the calculations (a-e). Blue (red) represents spin up (down). Note that in this material we have two different z coordinates for Cr atoms, where the dotted balls represent the ones that are lower in height.

We follow the same procedure for VOPc with a slight difference with respect to the case of [CpTi(cot)]. Depending on the calculated most stable site, the number of nearest neighbours can change. So, for *oxygen-up* we consider that the molecule is magnetically coupled to 4 Cr atoms whereas for *oxygen-down* we consider that it is coupled to 2 Cr atoms:

4.4) ***oxygen-up***

$$E_{AFM} - E_{FM} = -8J_4 S_{Cr} S_{Ti}$$

$$E_{AF1} - E_{FM} = -144 J_2 S_{Cr}^2$$

$$E_{AF2} - E_{FM} = (-72J_2 - 72J_3)S_{Cr}^2 - 4J_4 S_{Cr} S_{Ti}$$

$$E_{AF3} - E_{FM} = (-72J_1 - 72J_2)S_{Cr}^2 - 4J_4 S_{Cr} S_{Ti}$$

4.5) ***oxygen-down***

$$E_{AFM} - E_{FM} = -4J_4 S_{Cr} S_{Ti}$$

$$E_{AF1} - E_{FM} = -144 J_2 S_{Cr}^2$$

$$E_{AF2} - E_{FM} = (-72J_2 - 72J_3)S_{Cr}^2 - 2J_4 S_{Cr} S_{Ti}$$

$$E_{AF3} - E_{FM} = (-72J_1 - 72J_2)S_{Cr}^2 - 4J_4 S_{Cr} S_{Ti}$$

From such linear systems of equations we can extract the magnetic exchange parameters, which are shown in Table S6.

Table S6. Magnetic exchange parameters for pristine CrSBr and the [CpTi(cot)]@CrSBr and VOPc@CrSBr heterostructures.

		[CpTi(cot)]			VOPc	
	CrSBr	*standing$_{cot}$*	*standing$_{cp}$*	*lying*	*oxygen-up*	*oxygen-down*
J_1	2.93	2.82	2.77	2.77	2.66	2.65
J_2	3.55	3.59	3.60	3.59	3.55	3.55
J_3	2.33	2.78	2.68	2.72	2.05	2.00
J_4		0.6	0.26	0.03	-0.02	0.01

Table S7: Isotropic exchange parameters (meV) of the *standing$_{cot}$* heterostructure of [CpTi(cot)], distorted CrSBr and electron doped distorted CrSBr.

Magnetic exchange	*standing$_{cot}$*	Distorted CrSBr	Electron doped distorted CrSBr
J_1	2.82	2.63	2.80
J_2	3.59	3.54	3.58
J_3	2.78	1.81	3.14

The change in magnetic exchange after adsorption stems from (i) substrate distortion and (ii) charge transfer from molecule to substrate. To isolate substrate distortion effects, we estimated magnetic exchange in two cases: (i) distorted CrSBr (Figure S20, Table S7) and (ii) distorted CrSBr with electron doping (matching Bader charge transfer) in the *standing$_{cot}$* orientation. Structural distortion reduces J_3, while electron doping increases it compared to the heterostructure (Table S7). This discrepancy arises from the molecule-substrate exchange (J_4), which is absent in the electron-doped distorted CrSBr.

5. Calculation of the dipole-dipole interaction energy E_{dd}

The dipole-dipole interaction energy E_{dd} was calculated with the expression

$$E_{dd} = \sum_{S} \frac{\mu_0 \mu_b^2 g^2}{4\pi} \frac{3(\boldsymbol{S}_Q \cdot \boldsymbol{r}_S)(\boldsymbol{S} \cdot \boldsymbol{r}_S) - |\boldsymbol{r}_S|^5 \boldsymbol{S}_Q \cdot \boldsymbol{S}}{|\boldsymbol{r}_S|^5}$$

Here $\mathbf{S}_Q$ is the qubit spin, the summation is over all the Cr spins $\mathbf{S}$. $\mathbf{r}_s$ is the vector between the Cr spin and the qubit spin. g-factor is considered equal to 2 for all the spins. During the calculations the summation was cut at the distance of 3 CrSBr unit cells from the qubit spin.

6. Spin dynamics of qubit in [CpTi(cot)]@CrSBr and VOPc@CrSBr heterostructures

Further insights of playing with symmetry and coordination ligands should originate by analyzing the spin dynamics of the qubit after adsorption on CrSBr. This can be ascribed to the relaxation mechanism of qubit which is affected by low energy vibrational modes. Therefore, controlling the local vibration is a winning strategy for potential design of qubit that operate at higher temperature.

6.1. Unfolding magnon bands.

Introducing qubits increases the unit cell of the structure, leading to band folding. However, the additional spins have only moderate impact on the conventional acoustic and optical magnons in CrSBr across most of the spectrum. This allows us to isolate these bands, unfold them, and compare them with the magnon dispersion in pristine CrSBr as shown in Figure 4(a) of the main text.

The magnon energies, $\varepsilon_y(\boldsymbol{K})$, and magnon wavefunctions, $\Psi_{y;j}(\boldsymbol{K})$, for the supercells containing qubits were computed using RAD-tools[11] within the model described in the main text. Here, y enumerates the folded bands and j indexes magnetic atoms in the qubit-containing supercell, excluding the qubit itself. The wavevector $\boldsymbol{K}$ corresponds to the supercell and is defined within the limits:

$$K_x \in \left(-\frac{\pi}{Na}, \frac{\pi}{Na}\right), K_y \in \left(-\frac{\pi}{Mb}, \frac{\pi}{Mb}\right)$$

Here a and b are the unit cell parameters of CrSBr, and $N \times M$ supercell is assumed.
To establish the correspondence between the folded bands and the magnons in the pristine material, we performed an additional calculation using a single unit cell of CrSBr. In this calculation, we considered the exchange interactions J_1, J_2 and J_3 to be modified by qubits but excluded the qubits themselves, therefore, we did not include J_4. This yields energies $\varepsilon_x(\boldsymbol{k})$ and wavefunction $\varphi_{x;n}(\boldsymbol{k})$, where x indexes the unfolded bands (acoustical and optical magnons), and n enumerates Cr atoms in a single unit cell of CrSBr. The wavevector $\boldsymbol{k}$ lies within the reciprocal cell of CrSBr:

$$k_x \in \left(-\frac{\pi}{a}, \frac{\pi}{a}\right), k_y \in \left(-\frac{\pi}{b}, \frac{\pi}{b}\right).$$

For each band x, wavevector $\boldsymbol{k}$, and supercell atom j, we calculated the wavefunctions

$$\tilde{\Psi}_{x,j}(\boldsymbol{k}) = \varphi_{x;n(j)}(\boldsymbol{k}) \exp(-\boldsymbol{k} \cdot \boldsymbol{R}(j))$$

Here $n(j)$ is the unit cell atom corresponding to the supercell atom j. $\boldsymbol{R}(j)$ represents the position of the unit cell containing Cr atom j. Comparing the wavefunctions $\tilde{\Psi}_{x,j}(\boldsymbol{k})$ and $\Psi_{y;j}(\boldsymbol{K})$ allows us to determine which folded band y corresponds to the unfolded band x at wavevector $\boldsymbol{k}$. Here $\boldsymbol{K}$ is the wavevector of the folded bands corresponding to $\boldsymbol{k}$.

To quantify this correspondence, we define the following criterion:

$$C[\tilde{\Psi}_x(\boldsymbol{k})\Psi_y(\boldsymbol{K})] = \sum_j \left|\tilde{\Psi}_{x,j}(\boldsymbol{k})\left(\Psi_{y;j}(\boldsymbol{K})\right)^*\right|^2$$

Figure 3c in the main text shows the selection of y that maximizes C for each given $\boldsymbol{k}$ and x.

6.2. Qubit relaxation: theory.

We compute the shape of the single-magnon pulse using Wigner-Weisskopf theory.[16] We consider a single qubit with angular transition frequency ω_0 coupled to the magnon continuum. They are described by the Hamiltonian:

(1) $$\hat{H} = \hbar\omega_0|e\rangle\langle e| + \sum_{\boldsymbol{k}} \hbar\omega_{\boldsymbol{k}}\hat{s}_{\boldsymbol{k}}^{\dagger}\hat{s}_{\boldsymbol{k}} + \hbar\sum_{\boldsymbol{k}} (g_{\boldsymbol{k}}|e\rangle\langle g|\hat{s}_{\boldsymbol{k}} + \text{H.c.}) + \hat{H}_{noise}$$

Here $|e\rangle$ and $|g\rangle$ are the qubit excited and ground states, $\hat{s}_{\boldsymbol{k}}$ the bosonic annihilation operator for acoustic magnon mode **k**, with **k** the wavevector in Brillouin space and ω_k the magnon mode angular frequency. We consider only acoustic magnons because of the very high energy difference between the qubits and optical magnons. The third term in the above Hamiltonian describes magnon-qubit interactions where a magnon in mode **k** is either absorbed by exciting the qubit or emitted by qubit decay. These processes occur at a rate g_k controlled by exchange interaction J_4 between qubit spins and the spins of the neighboring Cr atoms

$$g_{\boldsymbol{k}} = \frac{\sqrt{3}}{2} J_4 \sum_n \frac{e^{i\boldsymbol{k}\boldsymbol{R}_n}}{\sqrt{L}}$$

Here n indexes the neighboring Cr atoms, $\boldsymbol{R}_n$ describes their positions, L is the total number of Cr atoms in the CrSBr layer.

The last term represents the noise experienced by both systems, namely the magnon damping and the qubit non-radiative decay, i.e. decay into channels other than magnons. They are described by the interaction of the respective systems with continuum thermal baths, i.e.,

(2) $$\hat{H}_{noise} = \hbar\int d\omega\omega\hat{Q}^{\dagger}(\omega)\hat{Q}(\omega) + \hbar\int d\omega\omega\sum_{\boldsymbol{k}}\hat{M}_{\boldsymbol{k}}^{\dagger}(\omega)\hat{M}_{\boldsymbol{k}}(\omega) + \hbar\sqrt{\frac{\gamma_{nr}}{2\pi}}\int d\omega[\hat{Q}(\omega)|e\rangle\langle g| + \text{H.c.}] + \hbar\sqrt{\frac{\gamma_m}{2\pi}}\int d\omega[\hat{M}$$

The first two terms represent the Hamiltonians of the bath causing qubit decay (with bosonic annihilation operator $Q(\omega)$, with ω a mute variable labelling the continuum of modes) and the baths causing damping of each magnon mode **k** (with bosonic annihilation operator $M_k(\omega)$). The third and fourth term represent the corresponding interactions with qubit and magnons. We assume that the associated interaction rates are frequency-independent and label them γ_{nr}(qubit non-radiative decay rate) and γ_m (magnon damping rate, assumed wavevector-independent for simplicity).
At low temperatures ($k_B T \ll \hbar\omega_0$, with T the temperature and k_B Boltzmann's constant) we can assume that by exciting the qubit one prepares an initial state where all the continuum baths (including magnons) have zero occupation, i.e. $|\psi(0)\rangle = |e\rangle|0\rangle|0\rangle|0\rangle$, where $|0\rangle$ represents the vacuum of each of the continuum baths. Since the total Hamiltonian conserves the number of quanta, the time-evolved wavefunction will remain in the single-excitation subspace, i.e. it will take the general form:

(3) $$|\psi(t)\rangle = c_e(t)|e\rangle|0\rangle|0\rangle|0\rangle$$
$$+\left(\sum_{\boldsymbol{k}} c_{\boldsymbol{k}}(t)\hat{s}_{\boldsymbol{k}}^{\dagger} + \int d\omega q(\omega,t)\hat{Q}^{\dagger}(\omega) + \int d\omega \sum_{\boldsymbol{k}} \mu_{\boldsymbol{k}}(\omega,t)\hat{M}_{\boldsymbol{k}}^{\dagger}(\omega)\right)|g\rangle|0\rangle|0\rangle|0\rangle$$

Dynamical equations for the unknown coefficients $\{c_e(t), c_k(t), q(\omega,t), \mu_k(t)\}$ can be derived by applying the Schrödinger's equation to the above state using the Hamiltonian (1). One obtains:

(4) $$\frac{d\tilde{c}_e}{dt} = -i\sum_{\boldsymbol{k}} g_{\boldsymbol{k}}\tilde{c}_{\boldsymbol{k}}e^{-i\Delta_{\boldsymbol{k}}t} - i\sqrt{\frac{\gamma_{nr}}{2\pi}}e^{i\omega_0 t}\int d\omega q(\omega)$$

(5) $$\frac{d\tilde{c}_{\boldsymbol{k}}}{dt} = -ig_{\boldsymbol{k}}^{*}\tilde{c}e^{i\Delta_{\boldsymbol{k}}t} - i\sqrt{\frac{\gamma_m}{2\pi}}e^{i\omega_{\boldsymbol{k}}t}\int d\omega\mu_{\boldsymbol{k}}(w)$$

(6) $$\frac{dq(\omega)}{dt} = -i\omega q(\omega) - i\sqrt{\frac{\gamma_{nr}}{2\pi}}c_e$$

(7) $$\frac{d\mu_{\boldsymbol{k}}(\omega)}{dt} = -i\omega\mu_{\boldsymbol{k}}(\omega) - i\sqrt{\frac{\gamma_m}{2\pi}}c_{\boldsymbol{k}}$$

Here we have defined the slowly oscillating variables $\tilde{c}_e(t) \equiv e^{i\omega_0 t}c_e(t)$ and $\tilde{c}_k(t) \equiv e^{i\omega_k t}c_k(t)$ and the magnon-qubit detuning $\Delta_k \equiv \omega_k - \omega_0$. One can formally solve the last two equations and insert them into the first two. Neglecting small magnon and qubit frequency renormalizations we obtain

(8) $$\frac{d\tilde{c}_e}{dt} = -\frac{\gamma_{nr}}{2}\tilde{c}_e - i\sum_{\boldsymbol{k}} g_{\boldsymbol{k}}e^{-i\Delta_{\boldsymbol{k}}t}\tilde{c}_{\boldsymbol{k}}$$

(9) $$\frac{d\tilde{c}_k}{dt} = -\frac{\gamma_m}{2}\tilde{c}_{\boldsymbol{k}} - ig_{\boldsymbol{k}}^{*}\tilde{c}_e e^{i\Delta_{\boldsymbol{k}}t}$$

We first obtain the qubit dynamics by formally integrating the second of these expressions and introducing it into the first, obtaining

$$(10) \quad \frac{d\tilde{c}_e}{dt} = -\frac{\gamma_{nr}}{2}\tilde{c}_e - \sum_{\boldsymbol{k}} |g_{\boldsymbol{k}}|^2 \int_0^t ds \tilde{c}_e(t-s) e^{-i\Delta_{\boldsymbol{k}} s} e^{-\frac{\gamma_m s}{2}}$$

Assuming that the qubit relaxation is Markovian, which is justified in [see section 5.3], we simplify this expression to

$$\frac{d\tilde{c}_e}{dt} \approx -\frac{\gamma_T}{2}\tilde{c}_e$$

This represents an exponential decay of the qubit occupation ($c_e = \exp\left(-\frac{\gamma_T t}{2}\right)$) with a total decay rate with a nonradiative contribution and a magnon contribution,

$$(11) \quad \gamma_T = \gamma_{nr} + \frac{\gamma_m ab}{4\pi^2} \int d\boldsymbol{k} \frac{\left|g_{\boldsymbol{k}}\sqrt{L}\right|^2}{\Delta_{\boldsymbol{k}}^2 + \left(\frac{\gamma_m}{2}\right)^2}$$

In the last expression we have taken the limit of an infinite magnonic substrate where the sum becomes an integral over the first Brillouin zone. We have assumed a rectangular lattice with lattice parameters a and b, and total number of cells L with $L \rightarrow \infty$ (note however that the quantity $\left|\sqrt{L} g_k\right|$ remains finite). Introducing the solution of the coefficient c_e into Eq. (10) we can obtain the amplitude of the emitted magnon in wavevector space,

$$(12) \quad c_{\boldsymbol{k}}(t) = \frac{i g_{\boldsymbol{k}}^*}{-i\Delta_{\boldsymbol{k}} + \left(\frac{\gamma_m - \gamma_T}{2}\right)} e^{(-i\omega_0 - \gamma_T/2)t}\left[e^{\left[-i\Delta_{\boldsymbol{k}} + \left(\frac{\gamma_T - \gamma_m}{2}\right)\right]t} - 1\right]$$

To find the shape of the single-magnon pulse in real space, we combine the definition of k-space magnon annihilation operators (hereafter we follow the notation of Ref. [17]),

$$(13) \quad \hat{b}_{nj} = \frac{1}{\sqrt{L}} \sum_{\boldsymbol{k}} \hat{b}_j(\boldsymbol{k}) e^{i\boldsymbol{k}\boldsymbol{r}_n}$$

And their expression in terms of the true Bogoliubov spin wave eigenmode operators,

$$(14) \quad \begin{bmatrix} \hat{b}_1(\boldsymbol{k}) \\ \hat{b}_2(\boldsymbol{k}) \\ \hat{b}_1^\dagger(-\boldsymbol{k}) \\ \hat{b}_2^\dagger(-\boldsymbol{k}) \end{bmatrix} = T(\boldsymbol{k}) \begin{bmatrix} \hat{s}_1(\boldsymbol{k}) \\ \hat{s}_2(\boldsymbol{k}) \\ \hat{s}_1^\dagger(-\boldsymbol{k}) \\ \hat{s}_2^\dagger(-\boldsymbol{k}) \end{bmatrix} = \begin{bmatrix} R(\boldsymbol{k}) & Q(\boldsymbol{k}) \\ Q^\dagger(-\boldsymbol{k}) & R^\dagger(-\boldsymbol{k}) \end{bmatrix} \begin{bmatrix} \hat{s}_1(\boldsymbol{k}) \\ \hat{s}_2(\boldsymbol{k}) \\ \hat{s}_1^\dagger(-\boldsymbol{k}) \\ \hat{s}_2^\dagger(-\boldsymbol{k}) \end{bmatrix}$$

Where in the last step we write the 4x4 Bogoliubov transformation matrix T in terms of 2x2 boxes R and Q. These matrices are obtained by diagonalizing the spin Hamiltonian

with rad-tools. Combining these expressions, we can write the average magnon number in each spin j within lattice site n,

$$(15)\quad \left\langle \hat{b}_{nj}^{\dagger}(t)\hat{b}_{nj}(t)\right\rangle = \frac{ab}{4\pi^2}\int d^2\boldsymbol{k}\int d^2\boldsymbol{q}e^{i\boldsymbol{r}_n(\boldsymbol{k}-\boldsymbol{q})}p_j(\boldsymbol{k},\boldsymbol{q})\left(g_{\boldsymbol{q}}\sqrt{L}\right)\left(g_{\boldsymbol{k}}^{*}\sqrt{L}\right)e^{-it(\omega_{\boldsymbol{k}}-\omega_{\boldsymbol{q}})}$$

$$\times\left(R_{j1}^{*}(\boldsymbol{q})R_{j1}(\boldsymbol{k})+Q_{j1}^{*}(-\boldsymbol{k})Q_{j1}(-\boldsymbol{q})\right)\left[\frac{e^{(i\Delta_{\boldsymbol{k}}-\Gamma_T/2)t}-e^{\frac{-\gamma_m t}{2}}}{i\Delta_{\boldsymbol{k}}+\frac{(\gamma_m-\Gamma_T)}{2}}\right]\left[\frac{e^{(-i\Delta_{\boldsymbol{q}}-\Gamma_T/2)t}-e^{\frac{-\gamma_m t}{2}}}{-i\Delta_{\boldsymbol{q}}+\frac{(\gamma_m-\Gamma_T)}{2}}\right]$$

The above expression is the quantity we compute and show in Figure 4 of the main text where we have considered magnon relaxation γ_m to be small.

6.3. Qubit Relaxation: Justification of the Markov Approximation

The Markov theory neglects the possibility of re-absorption of magnon emitted by the qubit. It is valid when either the magnon relaxation is faster than the qubit dynamics $\gamma_m >> \gamma_{nr}, |g_{\boldsymbol{k}}|$ or magnon transport in CrSBr is efficient enough to prevent the accumulation of magnon density at the Cr atoms interacting with the qubit spin.

In this section, we demonstrate that the second condition is satisfied in our case, ensuring the validity of the Markov approximation even for small γ_m. In this section we consider $\gamma_{nr} = \gamma_m = 0$, meaning all processes remain coherent. Under these conditions, the magnons at sites interacting with the qubit can be described by the wavefunction

$$(16)\quad c_n = \sum_{\boldsymbol{k}}\frac{c_{\boldsymbol{k}}e^{i\boldsymbol{k}\boldsymbol{R_n}}}{\sqrt{L}}$$

Where the magnon wavefunctions in momentum space $c_{\boldsymbol{k}}$ are estimated as

$$(17)\quad c_{\boldsymbol{k}} = \frac{ig_{\boldsymbol{k}}^{*}}{-i\Delta_{\boldsymbol{k}}+\left(\gamma_T/2\right)}e^{(-i\omega_0-\gamma_T/2)t}\left[e^{\left[-i\Delta_{\boldsymbol{k}}+\left(\gamma_T/2\right)\right]t}-1\right]$$

Figure MMM presents the absolute values of these wavefunctions $|c_n|$ for Cr spins interacting with the qubit under different conditions. Here γ_T is calculated with Eq. (11) with $\gamma_{nr} = \gamma_m = 0$. Our results show an initial increase in magnitude, followed by a gradual decrease accompanied by quantum oscillations. Crucially, in all cases, the calculated values remain small, $|c_n| << 1$, validating the applicability of the Markov approximation for describing magnon emission.

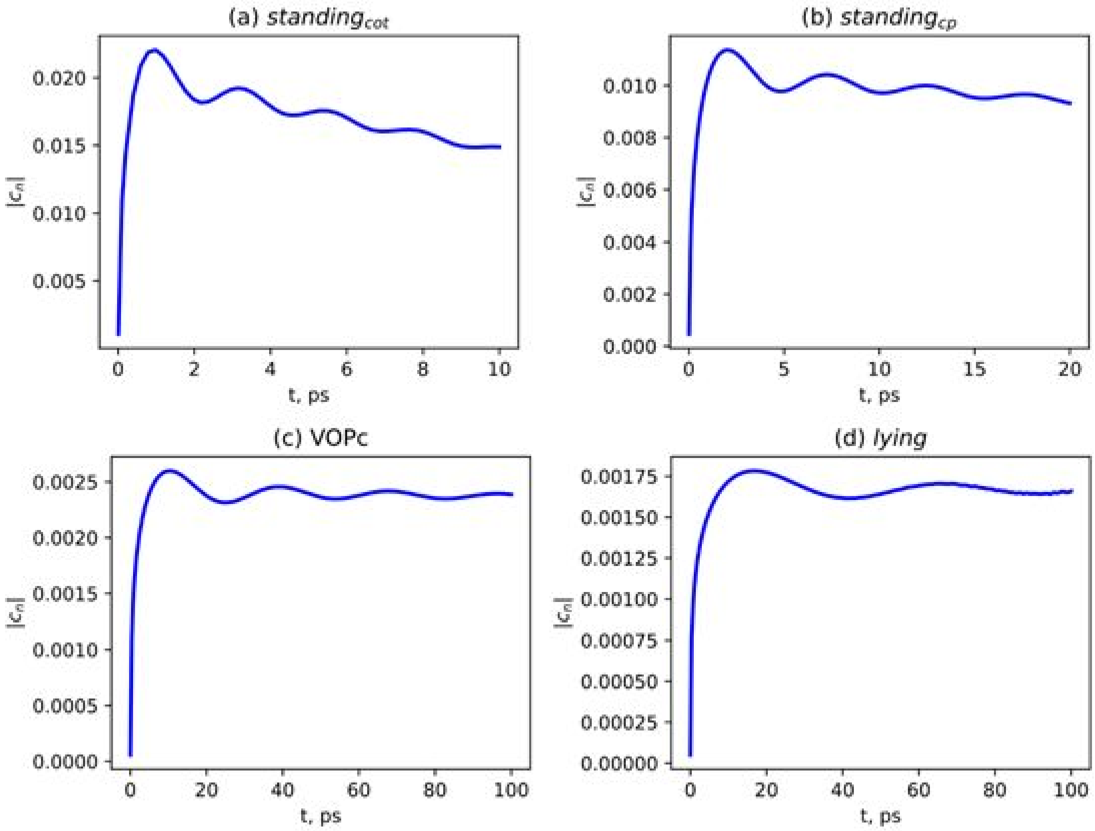

Figure S22. The absolute value $|c_n|$ of the magnon wavefunction on the Cr atoms interacting with qubits.

6.4. Qubit relaxation of [CpTi(cot)]@CrSBr and VOPc@CrSBr heterostructures in less favorable configurations.

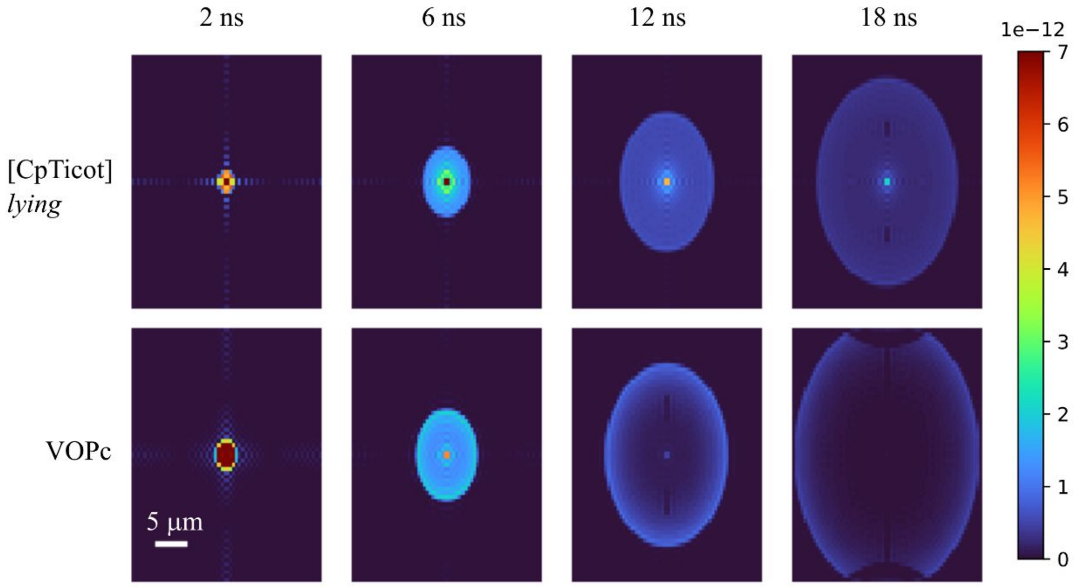

Figure S23. The magnon pulses emitted by the qubits [CpTi(cot)] in lying configuration (top) and VOPc in *oxygen-down* configuration (bottom) evolving on ns timescales and μm spatial scale.

6.5. Magnon group velocities

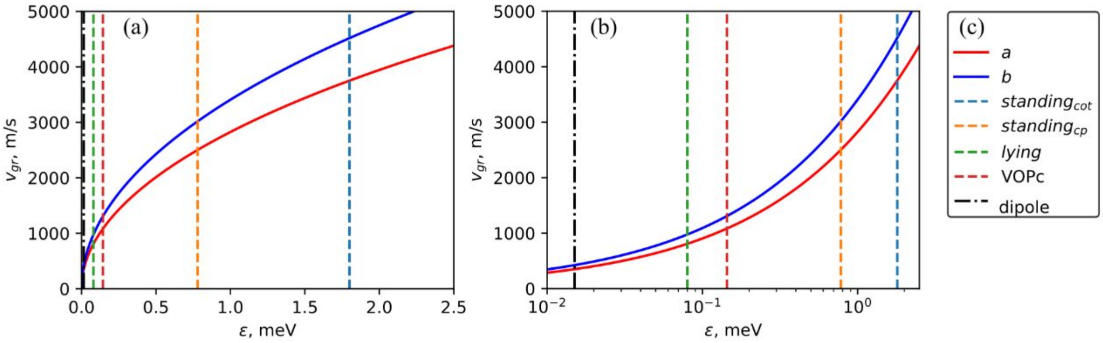

Figure S24. Magnon group velocities calculated for magnons propagating in *a*-direction (red solid curve) and *b*-direction (blue solid curve) as functions of magnon energy. Dashed lines show the energies of [CpTi(cot)] in $standing_{cot}$, $standing_{cp}$ and *lying* orientations and of *oxygen-up* VOPc qubit as specified in legend (panel (c)). Black dash-dotted line corresponds to a typical energy of dipole-dipole interaction of 0.015 meV. (a) corresponds to the linear scale for the energies and (b) for the logarithmic scale.

Magnon group velocities v_{gr} in pristine CrSBr are calculated as derivatives dε/dp of magnon energy over magnon momentum for magnon propagating in *a* and *b* direction. Fig S23 shows them as a function of magnon energy in comparison with the energies of the qubits considered in the article and with a typical energy of dipole-dipole interaction 0.015meV.

The group velocity corresponding to *standing*$_{cot}$ [CpTi(cot)] qubit (~4200 m/s) is ~3.5 times larger that the velocity corresponding to *oxygen-up* VOPc qubit (~1200 m/s) and ~11 times larger that the group velocity corresponding to the typical energy of dipole-dipole interaction (~370 m/s).

7. References

(1) Neese, F. Software Update: The ORCA Program System—Version 5.0. *WIREs Computational Molecular Science* **2022**, *12* (5). https://doi.org/10.1002/wcms.1606.

(2) Weigend, F.; Ahlrichs, R. Balanced Basis Sets of Split Valence, Triple Zeta Valence and Quadruple Zeta Valence Quality for H to Rn: Design and Assessment of Accuracy. *Physical Chemistry Chemical Physics* **2005**, *7* (18), 3297. https://doi.org/10.1039/b508541a.

(3) Neese, F.; Wennmohs, F.; Hansen, A.; Becker, U. Efficient, Approximate and Parallel Hartree–Fock and Hybrid DFT Calculations. A 'Chain-of-Spheres' Algorithm for the Hartree–Fock Exchange. *Chem Phys* **2009**, *356* (1–3), 98–109. https://doi.org/10.1016/j.chemphys.2008.10.036.

(4) Giannozzi, P.; Baroni, S.; Bonini, N.; Calandra, M.; Car, R.; Cavazzoni, C.; Ceresoli, D.; Chiarotti, G. L.; Cococcioni, M.; Dabo, I.; Dal Corso, A.; de Gironcoli, S.; Fabris, S.; Fratesi, G.; Gebauer, R.; Gerstmann, U.; Gougoussis, C.; Kokalj, A.; Lazzeri, M.; Martin-Samos, L.; Marzari, N.; Mauri, F.; Mazzarello, R.; Paolini, S.; Pasquarello, A.; Paulatto, L.; Sbraccia, C.; Scandolo, S.; Sclauzero, G.; Seitsonen, A. P.; Smogunov, A.; Umari, P.; Wentzcovitch, R. M. QUANTUM ESPRESSO: A Modular and Open-Source Software Project for Quantum Simulations of Materials. *Journal of Physics: Condensed Matter* **2009**, *21* (39), 395502. https://doi.org/10.1088/0953-8984/21/39/395502.

(5) Perdew, J. P.; Burke, K.; Ernzerhof, M. Generalized Gradient Approximation Made Simple. *Phys Rev Lett* **1996**, *77* (18), 3865–3868. https://doi.org/10.1103/PhysRevLett.77.3865.

(6) Head, J. D.; Zerner, M. C. A Broyden—Fletcher—Goldfarb—Shanno Optimization Procedure for Molecular Geometries. *Chem Phys Lett* **1985**, *122* (3), 264–270. https://doi.org/10.1016/0009-2614(85)80574-1.

(7) Grimme, S.; Antony, J.; Ehrlich, S.; Krieg, H. A Consistent and Accurate *Ab Initio* Parametrization of Density Functional Dispersion Correction (DFT-D) for the 94 Elements H-Pu. *J Chem Phys* **2010**, *132* (15). https://doi.org/10.1063/1.3382344.

(8) Liechtenstein, A. I.; Anisimov, V. I.; Zaanen, J. Density-Functional Theory and Strong Interactions: Orbital Ordering in Mott-Hubbard Insulators. *Phys Rev B* **1995**, *52* (8), R5467–R5470. https://doi.org/10.1103/PhysRevB.52.R5467.

(9) Henkelman, G.; Arnaldsson, A.; Jónsson, H. A Fast and Robust Algorithm for Bader Decomposition of Charge Density. *Comput Mater Sci* **2006**, *36* (3), 354–360. https://doi.org/10.1016/j.commatsci.2005.04.010.

(10) Holstein, T.; Primakoff, H. Field Dependence of the Intrinsic Domain Magnetization of a Ferromagnet. *Physical Review* **1940**, *58* (12), 1098–1113. https://doi.org/10.1103/PhysRev.58.1098.

(11) Rybakov, A. *RAD-tools*. https://rad-tools.org (accessed 2025-03-29).

(12) Swain, A.; Tiwari, R. K.; Khatua, M.; Rajaraman, G. Fluxionality Modulating the Magnetic Anisotropy in Lanthanoarene [(HCnRn)2Ln(II/III)] (n = 4–8) Single-Ion Magnets. *Inorg Chem* **2023**, *62* (24), 9552–9562. https://doi.org/10.1021/acs.inorgchem.3c00956.

(13) Sharma, T.; Singh, M. K.; Gupta, R.; Khatua, M.; Rajaraman, G. In Silico Design to Enhance the Barrier Height for Magnetization Reversal in Dy(Iii) Sandwich Complexes by Stitching Them under the Umbrella of Corannulene. *Chem Sci* **2021**, *12* (34), 11506–11514. https://doi.org/10.1039/D1SC03160K.

(14) Rivero-Carracedo, G.; Rybakov, A.; Baldoví, J. J. Magnon Sensing of NO, NO2 and NH3 Gas Capture on CrSBr Monolayer. *Chemistry – A European Journal* **2024**, *30* (51), e202401092. https://doi.org/https://doi.org/10.1002/chem.202401092.

(15) Ruiz, A. M.; Rivero-Carracedo, G.; Rybakov, A.; Dey, S.; Baldoví, J. J. Towards Molecular Controlled Magnonics. *Nanoscale Adv.* **2024**, *6* (13), 3320–3328. https://doi.org/10.1039/D4NA00230J.

(16) Scully, M. O. (Marlan O. *Quantum Optics*; Zubairy, M. Suhail., Ed.; Cambridge University Press: Cambridge, 1997.

(17) Toth, S.; Lake, B. Linear Spin Wave Theory for Single-Q Incommensurate Magnetic Structures. *Journal of Physics: Condensed Matter* **2015**, *27* (16), 166002. https://doi.org/10.1088/0953-8984/27/16/166002.
